# Identification of Extracellular Vesicle Signatures of Daratumumab Treated Multiple Myeloma

**DOI:** 10.1002/jev2.70213

**Published:** 2026-02-08

**Authors:** Kieran Brennan, Katrine F. Iversen, Alfonso Blanco‐Fernández, Thomas Lund, Torben Plesner, Margaret M. Mc Gee

**Affiliations:** ^1^ School of Biomolecular and Biomedical Science University College Dublin (UCD) Dublin Ireland; ^2^ Conway Institute of Biomolecular and Biomedical Research University College Dublin (UCD) Dublin Ireland; ^3^ Institute of Regional Health Science University of Southern Denmark Vejle Denmark; ^4^ Department of Internal Medicine, Section of Hematology, Lillebaelt Hospital University Hospital of Southern Denmark Vejle Denmark; ^5^ Flow Cytometry Core Technology, UCD Conway Institute of Biomolecular and Biomedical Research University College Dublin (UCD) Dublin Ireland; ^6^ Centre for Innovative Medical Technology Odense University Hospital Odense Denmark

**Keywords:** blood plasma, bone marrow plasma, daratumumab, EV biomarkers, extracellular vesicles, long‐term responders, multiple myeloma

## Abstract

Daratumumab (DARA) is a human monoclonal antibody for the treatment of multiple myeloma (MM), an incurable hematologic malignancy characterised by the accumulation of malignant plasma cells, located in the bone marrow (BM). We previously reported that peripheral blood plasma (PB) extracellular vesicles (EVs), isolated from 57 MM patients treated with DARA contain elevated CD55, CD59 and CD147 relative to healthy PB EVs, and elevated PDL1 was associated with patient response to DARA. The aim of this study was to identify additional proteins altered in these patients in order to generate predictive MM EV protein signatures. Flow cytometry analysis revealed that CD31, CD36 and CD44 were significantly elevated in MM PB EVs relative to healthy PB EVs, while CD8 and LAT1 were significantly decreased. CD38, LAT1 and PDL1 were significantly higher in PB EVs of patients with a long‐term response to DARA. Multivariate ROC curves revealed a diagnostic signature (MM panel) with a sensitivity 86.4% and specificity 91.6%, and a predictive signature (Response panel) with a sensitivity 80% and specificity 91.2%. In conclusion we identified two EV signatures that may have potential as a non‐invasive liquid biopsy to complement or replace invasive BM sampling for monitoring patient response to DARA.

## Graphical abstract

Multiple myeloma peripheral blood extracellular vesicles (EVs) are a source of non‐invasive biomarkers with potential for routine monitoring and to complement or replace invasive bone marrow biopsy. In this study, five proteins were significantly different from healthy controls and three associated with long‐term response on Daratumumab which may have diagnostic/prognostic value.

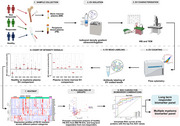

## 1 Introduction

Multiple myeloma (MM) is an incurable hematologic malignancy characterised by the accumulation of malignant plasma cells, usually located in the bone marrow (BM) (Kyle and Rajkumar 2008). Remarkable progress has been made in the treatment of MM with the introduction of immunomodulatory drugs, proteasome inhibitors, monoclonal antibodies, CAR T cells and T‐cell engager bispecific antibodies such as teclistamab, elranatamab; and talquetamab (for review see [Kegyes et al. 2023; Cirstea et al. 2025]). Daratumumab (DARA) is a CD38 antibody, and the first monoclonal antibody targeting CD38 introduced into clinical practice to treat MM; however, resistance eventually develops, and the disease progresses (de Weers et al. 2011). There is currently no reliable biomarker to predict which patients will respond to DARA with long‐term remission. Patients are likely to suffer relapse, due to the persistence of tumour cells after therapy, known as minimal residual disease (MRD) (Moreau et al. 2019; Avet‐Loiseau et al. 2021; Paiva et al. 2008). MRD testing in MM is increasingly being used in clinical trials for the assessment of disease response and as a prognostic tool for predicting response duration (Lahuerta et al. 2017; Munshi et al. 2017), with the FDA's Oncologic Drugs Advisory Committee (ODAC) in April 2024 supporting MRD‐negative complete response as an early endpoint reasonably likely to predict clinical benefit in MM that may be used to support an accelerated approval end point in clinical trials of MM (Landgren and Devlin 2025).

The main downside of MRD testing is the need for invasive BM aspirate, and that a single BM sampling might not accurately capture the full extent of the disease due to the spatial heterogeneity, as well as patchy involvement of MM, potentially leading to false negative results. Other biomarkers are primarily assessed at the time of diagnosis to assess risk, and that remains largely unchanged for the duration of the disease course, these include; albumin, beta‐2‐microglobulin, elevated LDH, high risk cytogenetic abnormalities and disease‐associated M‐protein. Extracellular vesicles (EVs) are promising liquid biopsy‐based biomarkers that can provide the opportunity for dynamic risk assessment, by tracking disease activity over time to indicate response to therapy. EVs are small membrane particles released by all living cells and mediate intercellular communication by carrying proteins, lipids and nucleic acids, while apoptotic bodies released during cell death are also considered EVs (Welsh et al. 2024). We previously characterized EVs from peripheral blood (PB) and BM from MM patients treated with DARA and compared the EV markers; CD9, CD63, CD81 and CD147, and CD38, PDL1, CD55 and CD59 between healthy controls and DARA‐responders with non‐responders. We found that CD55, CD59 and CD147 were elevated on MM PB EVs compared with healthy controls, and the level of PDL1 on MM PB EVs is higher in patients responding to treatment with DARA (Brennan et al. 2022). The aim of this study is to expand on this list of biomarkers in order to determine if a diagnostic signature can be developed that can distinguish between MM patients and healthy controls. Furthermore, with two additional years of follow‐up data, we investigate a predictive biomarker signature capable of predicting a long‐term response to DARA. Using a proteomic screen of a small cohort of 10 MM PB EVs and 10 healthy control EVs we identified eight proteins which were then evaluated by flow cytometry in a larger cohort of 61 MM patients and 12 healthy controls. We found that the eight proteins were present in all patient samples with CD8, CD31, CD36 and CD44, and LAT1 correlating with MM patient EVs versus healthy control EVs, while CD38, LAT1 and PDL1 are higher in patients with a sustained response to DARA. Using the expanded list of 16 proteins a diagnostic signature (MM panel) was generated with a sensitivity 86.4% and specificity 91.6%, and a predictive signature (Response panel) with a sensitivity 80% and specificity 91.2%.

## 2 Methods

### 2.1 Study Population and Sample Collection

As previously described in Brennan et al. (2022), 57 patients diagnosed with MM according to the IMWG guidelines and treated with a DARA‐containing regimen at the Departments of Haematology at either Vejle Hospital or Odense University Hospital, Denmark participated in the study (Rajkumar et al. 2014). PB was obtained from 19 of these patients who were responding to treatment with DARA and 38 who were progressing, with a corresponding BM aspirate being obtained from 19 of these patients with progression. Additionally, PB and BM was obtained from four patients with untreated, newly diagnosed multiple myeloma (NDMM) and PB from 12 healthy subjects were included as controls. The healthy donors were matched by age and sex with the MM population. Additional patient characteristics are included in Table S1. Participation was voluntary, and written informed consent was obtained from all subjects. Samples were obtained between December 2019 and May 2021. Data on patient characteristics and number of prior treatment lines were retrospectively obtained from the electronic medical files and registered in a designated Research Electronic Data Capture (REDCap) database (Harris et al. 2019, 2009). The study was approved by The Regional Committees on Health Research Ethics for Southern Denmark (S‐20170212). Platelet‐free plasma (PFP) was obtained by centrifuging PB and BM aspirate samples two times at 2500 × *g* at 4°C, for 15 min. Samples were stored at −80°C until EV isolation.

### 2.2 EV Isolation and Characterisation

EVs were isolated from PB and BM aspirate samples by density gradient ultracentrifugation and characterised by TEM and western blotting for EV markers TSG101 and CD63, and markers for soluble protein and lipoprotein particle contaminants; Albumin, APOB, APOA1 and APOE as described in Brennan et al. (2022). All ultracentrifugations were performed in Beckman Coulter rotors and ultracentrifuge tubes at 120,000 × *g* AVG in Beckman Coulter Optima L‐100 XP or Beckman Coulter Optima MAX‐XP ultracentrifuges, with centrifugation durations based on a ‘50 nm cut‐off size’ adjustment to the centrifugation duration for each rotor as described in Livshits et al. (2015), with additional 5 min added to allow the rotor to come up to speed (Livshits et al. 2015). EVs were resuspended in 200 µL residual PBS and stored at −80°C prior to analysis. These EVs were characterised in Brennan et al. (2022), with NTA and TEM analysis of a representative sample identifying particles in the expected size range and western blots showing TSG101 and CD63 are enriched, while albumin, and lipoprotein markers; APOB APOA1 and APOE are reduced (Brennan et al. 2022).

### 2.3 Antibodies and Reagents

Flow cytometry antibodies; anti‐CD8‐PE‐CY7 (1–25, clone RPA‐T8, 25‐0088‐42 Thermo Fisher Scientific [Waltham, MA, USA]), anti‐CD9‐PE (1–25, clone M‐L13, 555372 BD Bioscience), anti‐CD29‐PE (1–100, clone TS2/16, 568729, BD Bioscience), anti‐CD31‐PE (1–100, clone WM59, 555446 BD Bioscience), anti‐CD36‐PE (1–200, clone TR9, MA119772 Thermo Fisher Scientific [Waltham, MA, USA]), anti‐CD38‐FITC (1–200, clone CYT‐38F2, 1911229 CYTOGNOS), anti‐CD44‐PE (1–200, clone 515, 550989 BD Bioscience), anti‐CD55‐BV750 (1–200, clone A10, 750101 BD Bioscience), anti‐CD59‐APC (1–200, clone OV9A2, 17‐0596‐42 Thermo Fisher Scientific [Waltham, MA, USA]), anti‐CD61‐PE (1–200, clone VI‐PL2, 555754 BD Bioscience), anti‐CD63‐PE (1–100, clone H5C6, 12‐0639‐42 Thermo Fisher Scientific), anti‐CD81‐APC (1–25, clone JS‐81, 551112 BD Bioscience), anti‐CD147‐APC (1–400, clone MEM‐M6/1, A15706 Thermo Fisher Scientific [Waltham, MA, USA]), anti‐CD321‐PE (1–200, clone M.Ab.F11, 552556 BD Bioscience), and anti‐LAT1‐PE (1–100, clone BU53, NBP2‐50465PE, Novus Biologicals), anti‐PD‐L1‐PE‐CY7 (1–100, clone MIH1, 558017 BD Bioscience), IgG1 isotype control‐FITC (1–125, clone MOPC‐21, 554679 BD Bioscience), IgG1 isotype control‐PE (1–125, clone MOPC‐21, 559320 BD Bioscience), IgG2 ‐BV750 (1–200, clone G155‐178, 553456 BD Bioscience).

### 2.4 Flow Cytometric Analysis

Flow cytometry analysis was performed on the Beckman Coulter CytoFLEX LX Flow Cytometer as described in Brennan et al. (2022). 1.25 × 10^7^ EVs/test was mixed with 0.2 µL/test aldehyde/sulphate latex beads (4 µm; Thermo Fisher Scientific, Waltham, MA, USA) and beads were incubated in blocking buffer (1% BSA, 1 mM EDTA in PBS), followed by 100 mM glycine and FC block to prevent non‐specific protein binding. The beads were resuspended in blocking buffer and stained with antibodies for 30 min on ice. The samples were washed in PBS and flow cytometry analysis was performed on the Beckman Coulter CytoFLEX LX Flow Cytometer with gating performed based on FSC/SSC parameters. Since the EV samples were biobanked from the previous study some patients did not have enough EVs to test all proteins and the number of patients tested in each group is listed in Table S2. A detailed description of the flow cytometry analysis, including pre‐analytical and analytical procedures, is provided in the MIFlowCyt‐EV reports in Tables S3 and S4.

### 2.5 Mass Spectrometry Analysis

Mass spectrometry analysis was performed in duplicate on Bruker timsTof Pro mass spectrometer connected to an Evosep One liquid chromatography system as described in Brennan et al. (2022). For MS analysis, 2.5 × 10^8^ EV isolates (∼0.8 µg protein) were resuspended in 6 M urea, 50 mM Tris‐HCl, reduced and alkylated using dithiothreitol (8 mM final concentration) and iodoacetamide (20 mM final concentration). Then, samples were diluted to 1 M urea using 50 mM Tris‐HCl and digestion was continued overnight by the addition of sequencing grade modified trypsin (Promega, Madison, WI, USA, 1.5 µg trypsin/EV sample). The raw data were searched against the Homo sapiens subset of the UniProt/Swiss‐Prot Reviewed/DARA FASTA sequence using the search engine MaxQuant (release 2.0.1.0) using specific parameters for trapped ion mobility spectra data dependent acquisition (TIMS DDA). Each peptide used for protein identification met specific MaxQuant parameters. Specifically, only peptide scores that corresponded to a false discovery rate (FDR) of 0.01 were accepted from the MaxQuant database search. The normalised protein intensity of each identified protein was used for label‐free quantitation (LFQ).

### 2.6 Statistical Analysis

The medium fluorescent intensity (MFI) of each marker on the EVs is expressed as a median. The data sets were tested using the Mann–Whitney *U* test and Kruskal Wallis Test. *p* values of less than 0.05 were considered statistically significant. Heatmaps and principal component analysis (PCA) were performed using SRPLOT software (Tang et al. 2023). Receiver operating characteristic (ROC) and area under the curve (AUC) analysis was performed using Metaboanalyst 6.0 multivariate ROC curve based exploratory analysis tool, which uses an algorithm based on Monte–Carlo cross validation (MCCV) through balanced subsampling, coupled with a support vector machines (SVM) algorithm (Pang et al. 2024). In each MCCV, two‐thirds of the samples are used to evaluate feature importance. These features are then used to build appropriate classification models that are validated on the remaining one‐third of the samples. This analysis is repeated 100 times using different subsampling to calculate the performance and confidence interval (CI) for each model. All statistical analyses were performed using Statistics Kingdom Mann Whitney *U* test calculator and Kruskal Wallis Test Calculator (Statistics Kingdom 2017. Available from: http://www.statskingdom.com).

## 3 Results

### 3.1 Evaluation of the Additional Protein Markers in MM PB and BM EVs, and Healthy PB EVs

Brennan et al. found that CD55, CD59 and CD147 were present at higher levels in MM patients EVs, while PDL1 was present at higher levels in PB EVs from DARA responders. These EVs were isolated from either PB of MM patients and healthy controls and BM from DARA non‐responders by ultracentrifugation and iodixanol density gradient ultracentrifugation. These EV samples were found to contain the EV proteins CD9, CD63, CD81 and CD147 by flow cytometry and CD63 and TSG101 by western blot, while the levels of APOA1, APOB, APOE and albumin were depleted in the EV fraction relative to other gradient fractions in line with the MISEV guidelines (Brennan et al. 2022, Figure S1). This study aims to build on our previous work by identifying additional biomarkers in the same EV samples as Brennan et al. while making use of additional 2 years of patient follow‐up in order to look at short‐term versus long‐term response to DARA. The proteomic data from Brennan et al. was reanalysed in order to expand on these potential MM biomarkers, with the aim of identifying an EV signature of MM and patient response to DARA (Brennan et al. 2022). The proteomic data were filtered to include only membrane proteins that would be at the cell/EV surface and where antibodies were available for flow cytometry analysis. The data was further shortlisted to focus on membrane proteins that were significantly correlated with either MM patients with a greater than two‐fold increase in MM PB EVs relative to healthy PB EVs, as well as proteins that were significantly correlated with DARA response, with greater than 1.5‐fold change in responders or non‐responders (<0.66 or >1.5‐fold change) (Table S5). CD8, CD29, CD31, CD61, CD88, CD321 and LAT1 were selected from Table S5 for flow cytometry analysis in the patient cohort based on their being prior evidence for increased protein expression in cancer (Table S5). CD44 and CD321 were included in the panel as they have been previously associated with MM (Bjorklund et al. 2014; Solimando et al. 2018). CD88 was removed from the panel as the selected antibody recognised CD88 on cell samples but gave no signal on corresponding EV samples (data not shown).

**FIGURE 1:**
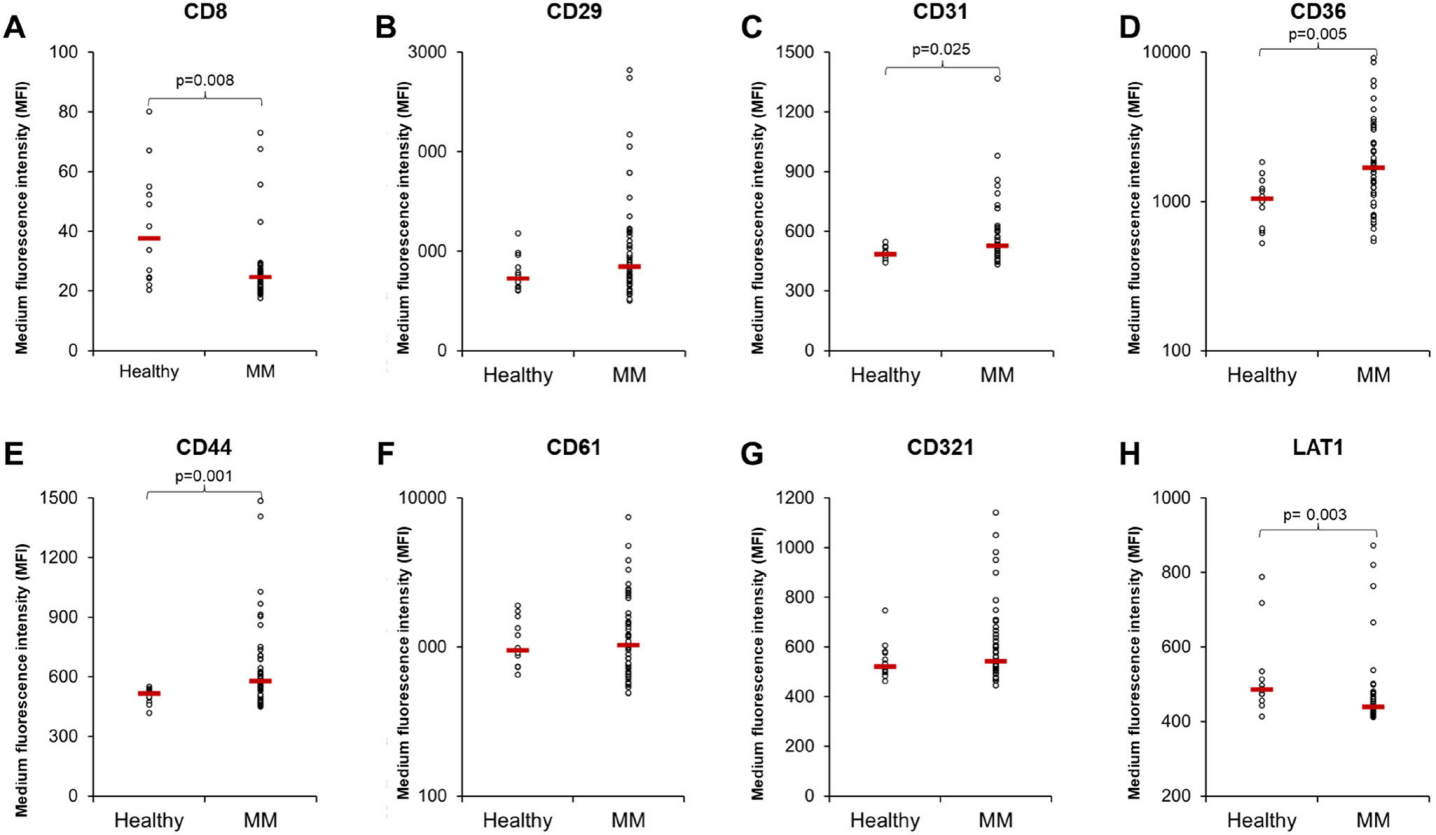
**Multiple myeloma peripheral blood EVs are positive for all markers and have elevated levels of CD31, CD36 and CD44, and decreased CD8 and LAT1 levels compared to healthy peripheral blood EVs**. Flow cytometry analysis of 1.25 × 10^7^ EVs/test from 49 to 54 MM patients (the number of patients tested for each protein is listed in Table S2) and 12 healthy controls were bound to the surface of 4 µm aldehyde/sulphate latex beads and stained with antibodies; CD8‐PE‐CY7, CD29‐PE, CD31‐PE, CD36‐PE, CD44‐PE, CD61‐PE, CD321‐PE and LAT1‐PE for 30 min on ice.

We first sought to evaluate the eight‐protein marker panel in MM PB and BM EVs, and Healthy PB EVs by flow cytometry in the patient cohort (54 MM PB patients [17 responders, 33 non‐responders and four NDMM] vs. 12 healthy PB controls; and 21 BM samples from non‐responders) as the original proteomic analysis was performed on a small subset of the patient cohort (10 MM patients [five responders and five non‐responders] vs. 10 healthy controls). Our flow cytometry results confirm that the eight protein markers identified by proteomic analysis of our small patient cohort were present in all PB EVs samples, validating our approach. CD31, CD36 and CD44 were significantly elevated in MM PB EVs compared to healthy PB EVs, while CD8 and LAT1 were present at significantly lower levels on MM PB EVs compared to healthy PB EVs (Figure 1). MM PB EVs had a higher level of CD29 (median MFI of 847 vs. 725.4) and CD321 (median MFI of 543 vs. 521.1) but it was not significantly different from healthy PB EVs, *p* = 0.14 and 0.26, respectively (Figure 1). There was no overall difference in CD61 between MM PB EVs compared to healthy PB EVs (Figure 1F).

**FIGURE 2:**
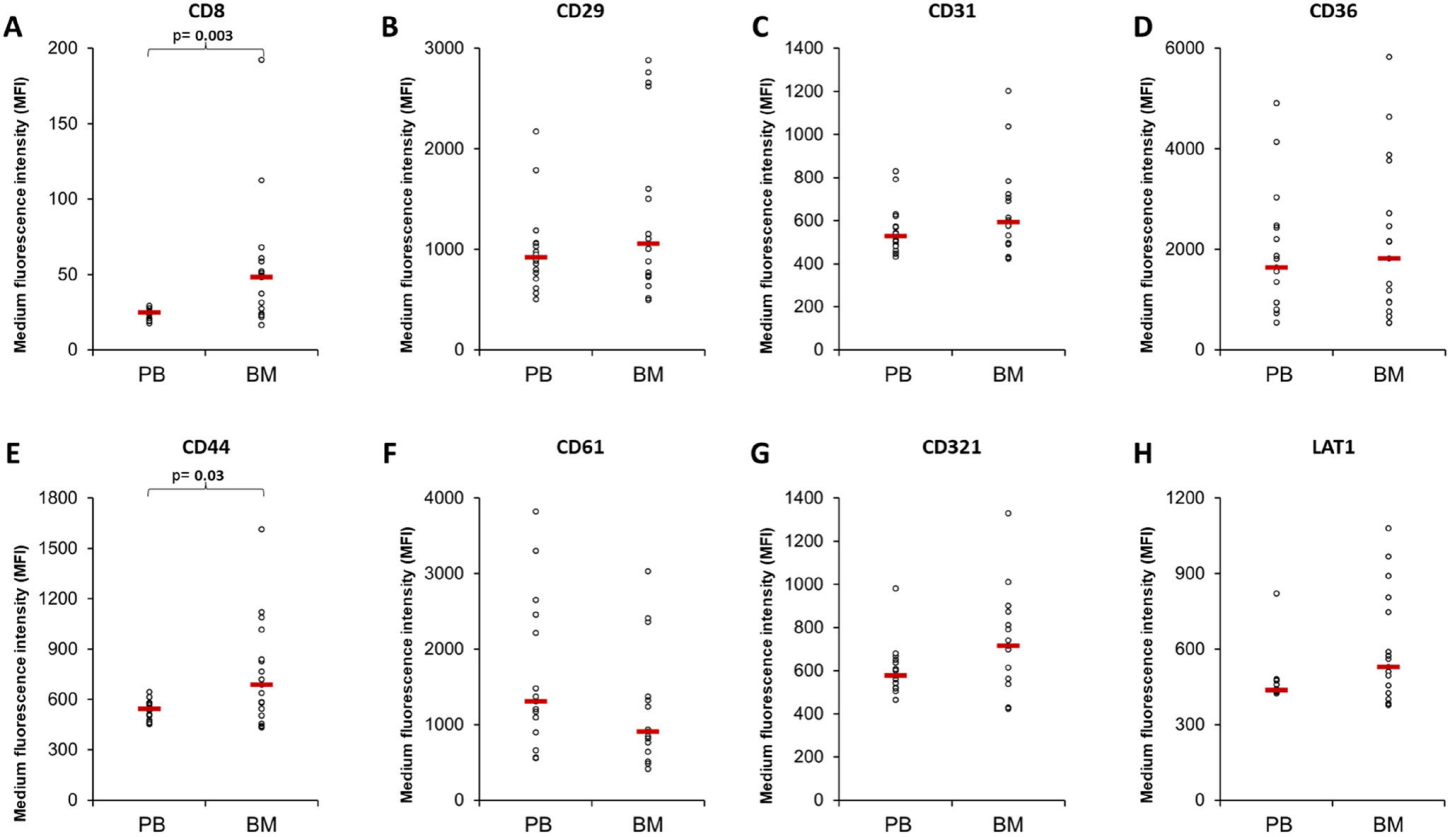
**Multiple myeloma bone marrow EVs are positive for all markers and have elevated CD8 and CD44 levels compared to peripheral blood EVs**. Flow cytometry analysis of 1.25 × 10^7^ EVs/test from matched BM and PB EVs from patients progressing on DARA were bound to the surface of 4 µm aldehyde/sulphate latex beads and stained with antibodies; CD8‐PE‐CY7, CD29‐PE, CD31‐PE, CD36‐PE, CD44‐PE, CD61‐PE, CD321‐PE and LAT1‐PE for 30 min on ice. Medium fluorescent intensity levels were normalised to average intensity of the EV markers CD9 and CD63 on each patient's matched peripheral blood and bone marrow EVs (*n* = 16 for CD61 and CD321; *n* = 17 for CD29; *n* = 18 for CD8, CD31, CD36 and LAT1; *n* = 19 for CD44).

PB is a useful tool for non‐invasive sampling as it allows for routine patient monitoring of treatment response relative to the gold standard of invasive BM sampling. However, the PB EVs population will contain EVs from all over the body and it is not possible to accurately determine the EV origin or the percentage of EVs that originate from the patient's myeloma cells. Since MM is characterized by monoclonal expansion of plasma cells in the BM, we hypothesise that EVs from MM cells would make up a larger portion of the BM EV population relative to the PB EV population. This is supported by our previous observation that there was an increased abundance of CD59 and CD147 on MM BM EVs relative to MM PB EVs (Brennan et al. 2022). We discovered that all eight proteins were expressed in MM BM samples which is the first time these proteins have been reported in MM BM EV samples. To allow for comparison between MM PB EVs and MM BM EVs the MFI for each marker was normalised to the average CD9/CD63 for each patient, with only patients with both a BM and PB sample being included in the comparison (Figure 2). CD8 (median MFI = 48/24.7) and CD44 (median MFI = 687/543.7) were present at significantly higher levels on BM EVs compared to matched PB EVs, *p* = 0.003 and *p* = 0.03, respectively (Figure 2). BM EVs had a higher level of LAT1 (median MFI = 529/436.8), CD61 (median MFI = 1309.3/905) and CD321 (median MFI = 715/577.7) but it was not significantly different from PB EVs, *p* = 0.099, *p* = 0.16 and *p* = 0.126, respectively (Figure 2). There was no overall difference in CD29, CD31 and CD36 levels on BM EVs compared to matched PB EVs (Figure 2). Since BM samples were only available for non‐responder patients as part of normal treatment we don't have access to healthy control BM EVs for comparison, therefore it is worth keeping in mind that we observed that CD8 is significantly lower in MM PB EVs and it is not possible to determine if CD8 is at normal levels or elevated in the BM of non‐responding MM patients relative to healthy controls.

### 3.2 The Association of the 16 Protein Panel With a Sustained Response to DARA

**FIGURE 3:**
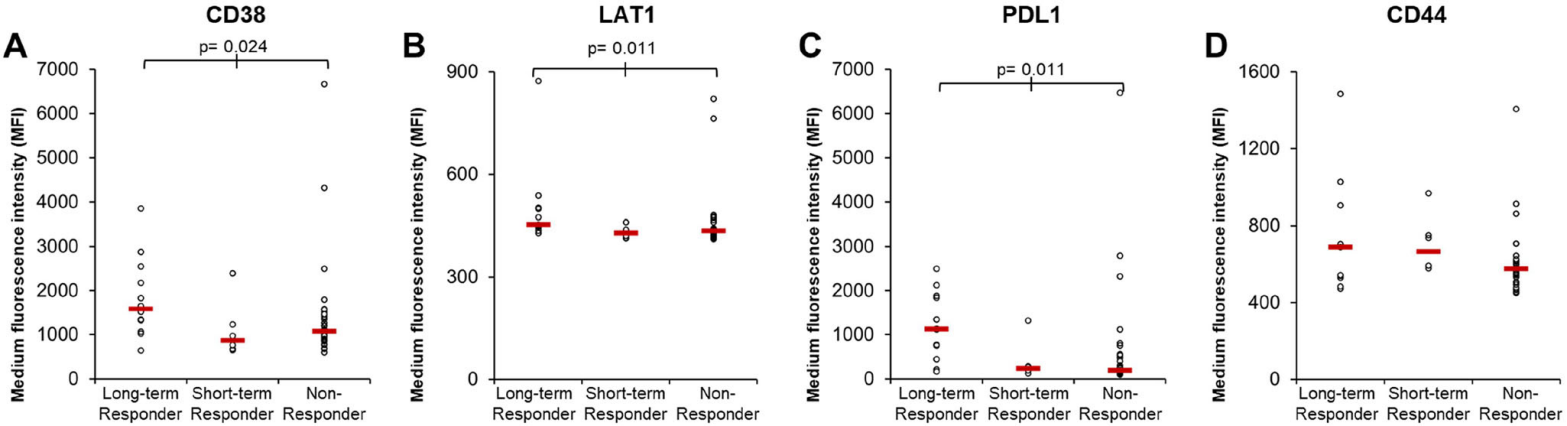
**CD38, LAT1 and PDL1 are higher on MM PB EVs from patients with a sustained response to DARA**. Flow cytometry analysis of 1.25 × 10^7^ EVs/test from 11 to 12 long‐term responders and 32–38 non‐responders (the number of patients tested for each protein is listed in Table S2) and six short‐term responders, were bound to the surface of 4 µm aldehyde/sulphate latex beads and stained with antibodies; CD38‐FITC, CD44‐PE, LAT1‐PE and PDL1‐PE‐CY7 for 30 min on ice.

Brennan et al. reported PDL1 at higher levels in the PB EVs of MM patients responding to a DARA‐containing regimen with a partial response or better, at the time of sample collection (Brennan et al. 2022). Since that study was performed there is now 2+ years additional follow‐up on the patients, which allowed patients to be subdivided into long‐term responders and patients who later progressed on DARA (short‐term responders). Using this follow‐up data the eight proteins examined in Brennan et al. (CD9, CD38, CD55, CD59, CD63, CD81, CD147 and PDL1) and the new eight protein panel revealed through this study (CD8, CD29, CD31, CD36, CD44, CD61, CD321 and LAT1) were examined to determine if EV protein levels could predict long‐term response to a DARA‐containing regimen. Three proteins (CD38, LAT1 and PDL1) were associated with a long‐term response to DARA (Figure 3). CD38 was significantly higher in PB EVs of patients with a long‐term response to DARA (median MFI = 1584.1) compared to the PB EVs of non‐responders (median MFI = 1076) and short‐term responders (median MFI = 864.9), *p* = 0.024 (Figure 3A). LAT1 was significantly higher in PB EVs of patients with a long‐term response to DARA (median MFI = 453.8) compared to the PB EVs of non‐responders (median MFI = 436) and short‐term responders (median MFI = 428.6), *p* = 0.011 (Figure 3B). PDL1 was significantly higher in PB EVs of patients with a long‐term response to DARA (median MFI = 1128) compared to the PB EVs of non‐responders (median MFI = 194) and short‐term responders (median MFI = 237.3), *p* = 0.003 (Figure 3C). CD44 was higher in initial responders (long‐term responders [median MFI = 689] and short‐term responders [median MFI = 665.7]); however, this was not significantly different from non‐responders (median MFI = 577), *p* = 0.127 (Figure 3D). It was also observed that CD44 was higher in DARA‐treated patients (median MFI = 579.6/487), compared to NDMM, *p* = 0.015 (Figure S2A). This is consistent with reports that CD44 is associated with Dexamethasone and Lenalidomide resistance in MM (Bjorklund et al. 2014; Ohwada et al. 2008). However, it should be noted that the sample size of patients with NDMM is only four patients and a future study with a larger number of patients would be needed to determine if this is a true finding. Finally, there was no difference in the amount of CD8, CD9, CD29, CD31, CD36, CD55, CD59, CD61, CD63, CD81, CD147 and CD321 on PB EVs of patients with a long‐term response to DARA compared to the PB EVs of short‐term responders and non‐responders (Figure S3).

### 3.3 The Creation of a Biomarker Signature for Myeloma and DARA Response Monitoring

**FIGURE 4:**
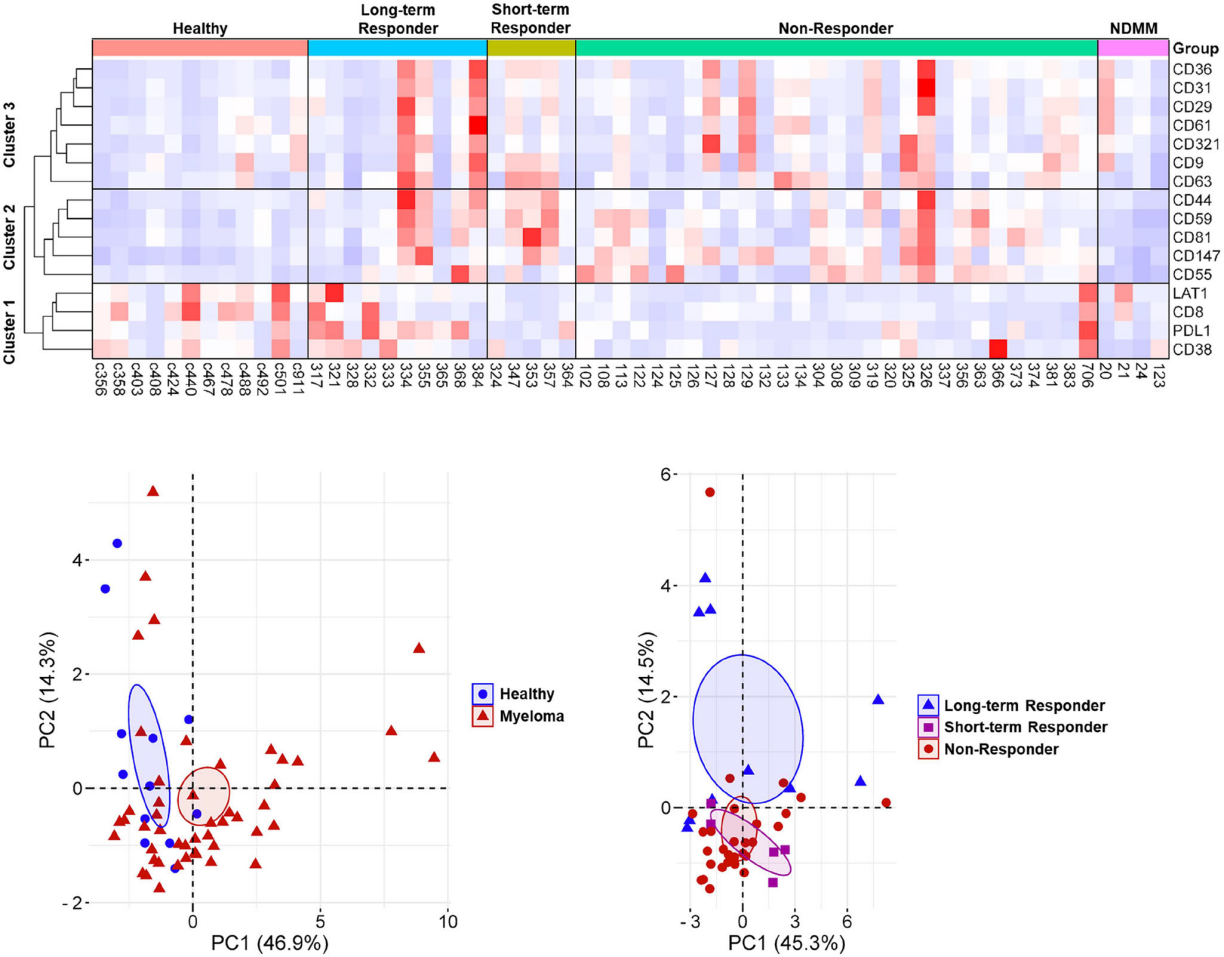
Expression profiles of PB EV markers across different patient categories. (**A**) Heatmap hierarchical clustering analysis of PB EV markers in patient categories (healthy, long‐term responder, short‐term responder, non‐responder and NDMM), with patient categories on the horizontal, and the 16 proteins on the vertical. Principal component analysis (PCA) of (B) healthy PB EVs from myeloma PB EVs, and (C) long‐term responders from short‐term responders and non‐responders. Only patients with enough sample quantity to detect all 16 markers were included in the analysis.

Our individual analysis of the eight protein markers in PB EVs revealed that three proteins (CD31, CD36 and CD44) were significantly elevated in MM PB EVs, while two proteins (CD8 and LAT1) were significantly decreased on MM PB EVs compared to healthy PB EVs (Figure 1). Furthermore LAT 1 was significantly associated with a long‐term response to DARA (Figure 3). In order to develop a biomarker signature for myeloma and second signature for DARA response monitoring, we next examined the association between different patient categories (healthy, long‐term responder, short‐term responder, non‐responder and NDMM) and the expression of all protein markers regardless of whether they were individually significantly associated with one group or not. This is because some markers may not be significant alone, but the combined expression of two or more markers can either increase the number of true positives detected or reduce the number of false positives allowing for improved marker performance. To develop a biomarker signature, the expression of the eight proteins from this study was combined with the data on the eight proteins from Brennan et al. (2022) to create a dataset of 16 proteins. Only patients with data from all 16 markers being included in the analysis (48 MM PB patients [10 long‐term responder, five short‐term responder, 29 non‐responders and four NDMM] vs. 12 healthy PB controls). To investigate further how different patient categories might share common causal mechanisms, we developed a heatmap, based on hierarchical clustering of the protein markers, with patient categories on the horizontal, and the 16 proteins on the vertical (Figure 4A). The heatmap revealed that the expression of proteins grouped into three main clusters, proteins in cluster 1 (CD8, CD38, LAT1 and PDL1) were higher in healthy and long‐term responders, while proteins in cluster 2 (CD44, CD55, CD59, CD81 and CD147) and 3 (CD9, CD29, CD31, CD36, CD61, CD63 and CD321) were lower in healthy controls, with cluster 2 also being lower in NDMM patients (Figure 4A). Since the heatmap has not been filtered by significance and instead is showing the expression of all proteins in all patients, these clusters may not be significant but they do highlight that different trends in marker abundance exist across the patient groups. Next, we conducted a PCA which revealed a separation between healthy PB EVs from MM PB EVs (Figure 4B), and long‐term responders from short‐term responders and non‐responders (Figure 4C) which supported the observations from the heatmap. Since the PCA analysis identified a separation in healthy PB EVs from MM PB EVs and long‐term responders PB EVs from other MM PB EVs using only two markers, we then expanded the marker selection using Metaboanalyst 6.0 multivariate ROC curve based exploratory analysis tool, which uses an algorithm based on MCCV through balanced subsampling, coupled with a SVM algorithm (Tang et al. 2023). Since we did not have a separate validation cohort for each MCCV, two‐thirds of the samples are used to evaluate feature importance (individual marker or marker panel MFI). These features are then used to build appropriate classification models that are validated on the remaining one‐third of the samples. This analysis is repeated 100 times using different subsampling to calculate the performance and confidence interval (CI) for each model.

### 3.4 Diagnostic Performance of the PB EV Markers in DARA‐Treated MM Patients

**FIGURE 5:**
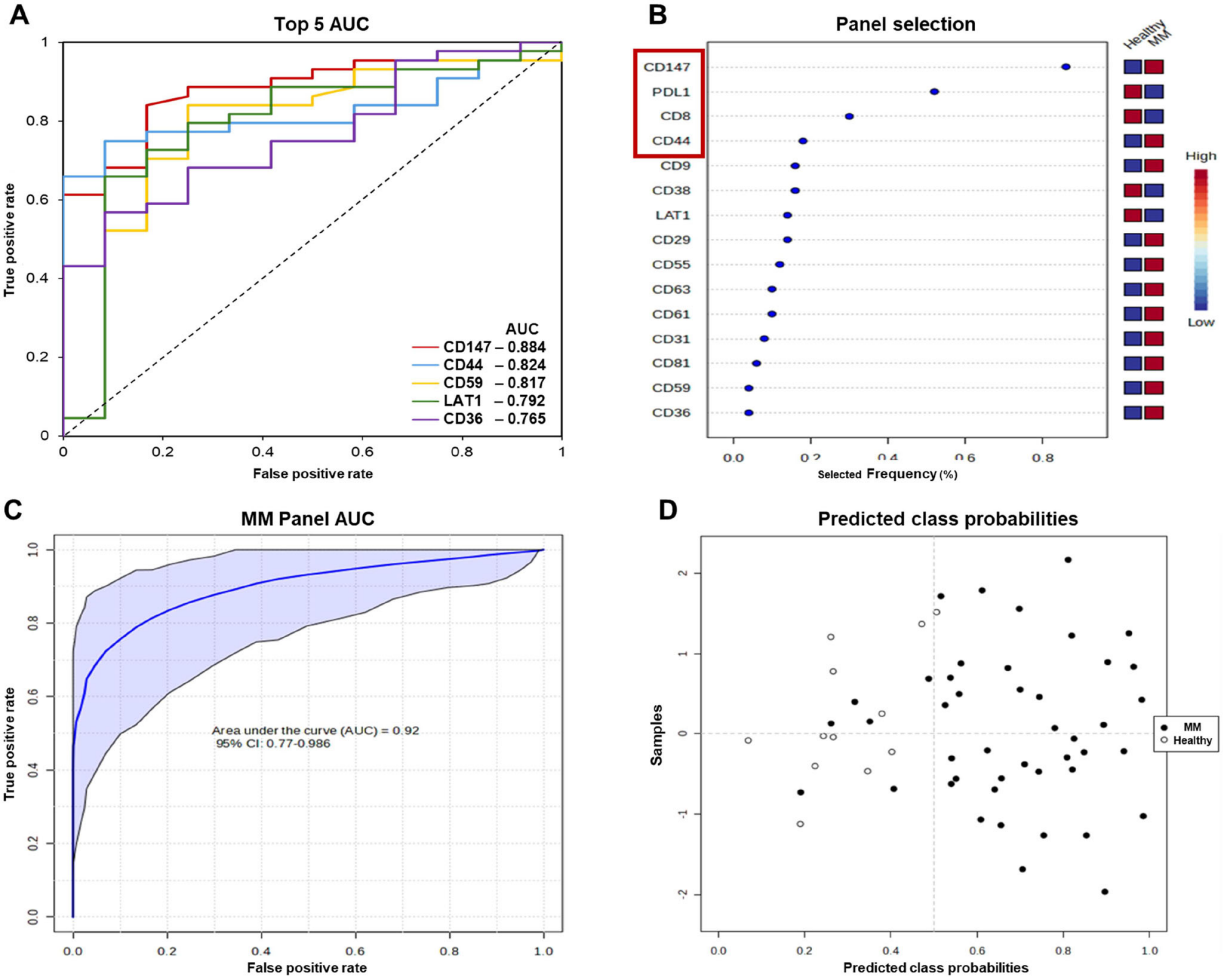
**Evaluation of the combined diagnostic efficacy of the MM panel to distinguish MM patients from healthy controls**. (A) Overlay of the Univariate ROC curves of the proteins with the top five AUC values. (B) Top 15 significant features ranked based on their frequencies of being selected during cross validation, with the combinations of biomarkers used for the MM panel in the red square. The frequency of being selected shows the stability of the rank of the importance for a given biomarker. (C) Multivariate ROC curve for the MM panel with the 95% CI shown in blue. (D) The average of predicted class probabilities of each sample across the 100 cross‐validations.

MM diagnosis currently requires invasive BM examination due to the lack of biomarker signatures. Therefore, the Metaboanalyst 6.0 multivariate ROC curve based exploratory analysis tool was used to assess the diagnostic performance of the PB EV markers in detecting DARA‐treated MM patients. First, univariate ROC curves were plotted to evaluate the diagnostic efficacy of each PB EV marker in DARA‐treated MM patients individually. AUC values for the PB EV markers ranged between 0.511 and 0.884 (Table 1), with the top five markers overlaid for comparison (Figure 5A).

**TABLE 1:** Univariate ROC curve statistics for MM patients relative to healthy controls.

Name	AUC	*t*‐tests	Log2 FC
**CD147**	0.88352	**0.0007**	−0.2644
**CD8**	0.74242	**0.0011**	0.64724
**CD59**	0.81723	**0.0041**	−0.5688
**CD55**	0.74242	**0.0111**	−0.7128
**CD81**	0.73106	**0.0259**	−0.6721
**CD44**	0.82386	**0.0267**	−0.3674
**CD36**	0.76515	**0.0344**	−1.1283
**LAT1**	0.79167	**0.0360**	0.1868
**CD63**	0.64394	0.0785	−0.4097
**CD31**	0.70265	0.0819	−0.24
**CD29**	0.64394	0.1143	−0.4085
**CD321**	0.60227	0.2038	−0.1593
**CD61**	0.51136	0.3689	−0.3901
**CD9**	0.54735	0.3807	−0.3132
**CD38**	0.62689	0.4323	0.24444
**PDL1**	0.55114	0.6166	−0.2849

Nine proteins had an AUC greater than 0.7, with eight proteins being significant (Table 1). To determine which of these markers would perform best together in an unbiased manner we used the Metaboanalyst 6.0 multivariate ROC curve based exploratory analysis (explorer) tool, with linear SVM as the classification method and SVM built‐in as the feature ranking method. To estimate the predictive performance as well as the stability of the selected features, a balanced Monte‐Carlo cross‐validation (MCCV) procedure with 50 iterations is used. In each MCCV, two‐thirds of the samples are randomly selected to evaluate the feature importance and the most important features are selected with different cutoffs to build models which are validated on the remaining 1/3 of the samples. Six biomarker models were created using a linear SVM with different numbers of markers (a combination of either 2, 3, 5, 7, 10 or 16 markers) to identify the model with the highest AUC. During cross‐validation (CV) not only are the optimal subset of markers being selected, the optimal MFI values for the associated modelling procedure are also estimated. In particular, each of the 50 iterations of the CV process produces different model parameter values, and hence potentially a different range of model prediction values. Figure 5B shows the important markers associated with these models based on their frequency of being selected in each iteration, and the red box highlights the PB EV markers selected to form the MM panel; CD8, CD44, CD147 and PDL1. To avoid overfitting a biomarker panel to this current dataset the number of biomarkers was limited to four as the remaining markers had a similar selected frequency and did not add a substantial improvement in AUC when added to the MM panel.

The MM panel was evaluated using the Metaboanalyst 6.0 ROC curve‐based model evaluation (Tester) tool, with linear SVM as the classification method and SVM built‐in as the feature ranking method. The tester tool performs 100 cross validations to produce a smooth ROC curve (Figure 5C) revealing that the MM panel has an AUC of 0.92 [95% CI 0.77–0.986, *p* < 0.001; Figure 5C], which is higher than the best performing individual marker, CD147 [AUC of 0.862, 95% CI 0.385–0.958, *p* = 0.001; Figure S4A]. The average of predicted class probabilities of each sample across the 100 CVs with the MM panel resulted in 38 of 44 MM patients, and 11 of 12 healthy controls being correctly classified into their respective groups (sensitivity 86.4% and specificity 91.6%), resulting in an overall accuracy of 87.5% (Figure 5D, Table 2), which is an improvement of CD147 accuracy alone of 71.4% (Figure S4B, Table 2). Since the algorithm uses a balanced sub‐sampling approach, the classification boundary is located at the centre of the plot (x = 0.5, the dotted line), while the y‐axis is arbitrary and functions to ensure all samples are visible.

**TABLE 2:** MM panel versus CD147 confusion matrix.

	MM panel		CD147	
	Negative	Positive	Negative	Positive
**Healthy**	11	1	11	1
**DARA‐treated MM**	6	38	15	29
**Accuracy**	87.5%		71.4%	
**Sensitivity**	86.4%		65.9%	
**Specificity**	91.7%		91.7%	
**Positive PV**	97.4%		96.7%	
**Negative PV**	64.7%		42.3%	

### 3.5 PB EV Markers as a Predictive Biomarker of DARA Response

**FIGURE 6:**
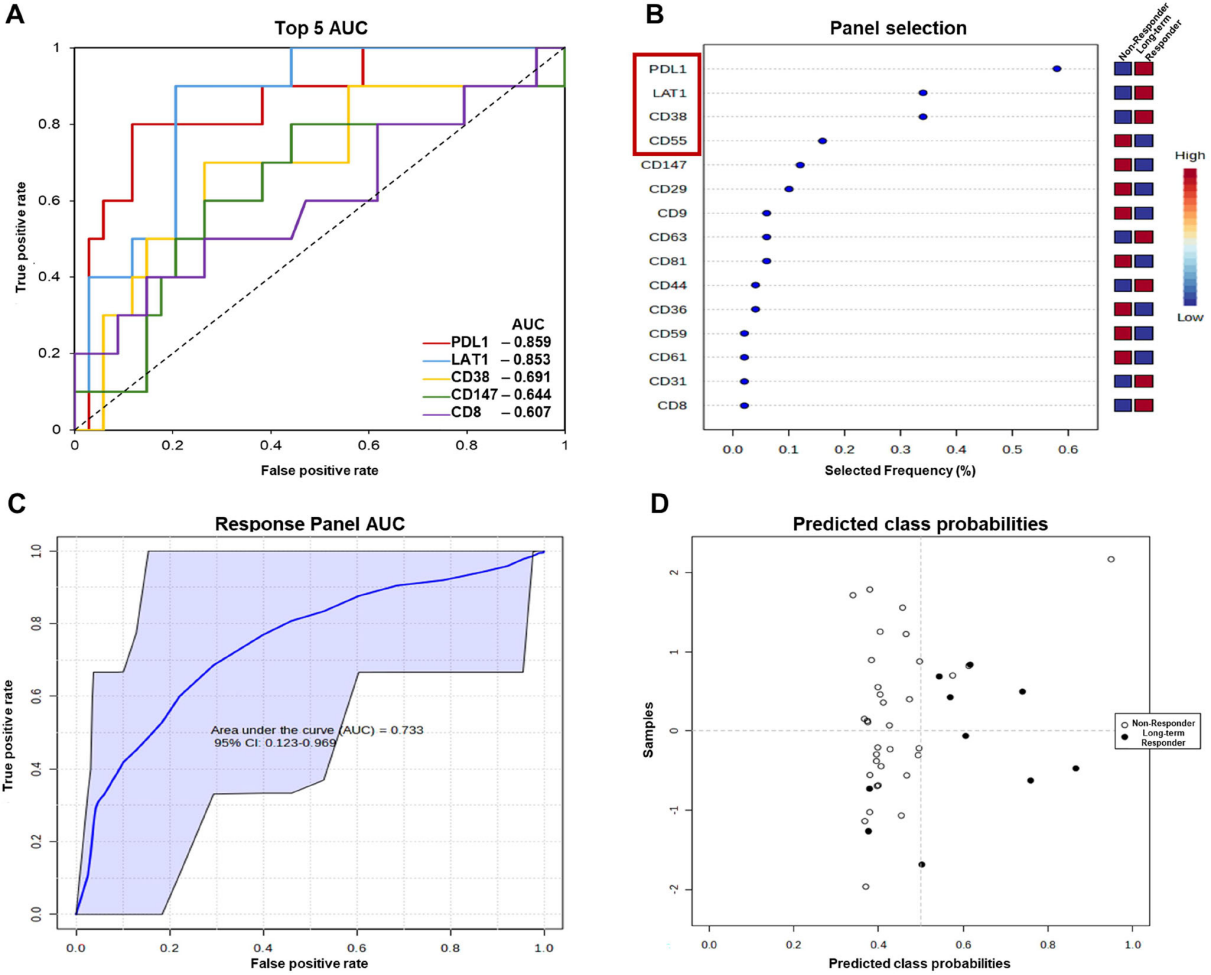
**Evaluation of the predictive efficacy of the DARA response panel to distinguish DARA long‐term responders from short‐term responders and non‐responders**. (A) Overlay of the univariate ROC curves of the proteins with the top five AUC values. (B) Top 15 significant features ranked based on their frequencies of being selected during cross validation, with the combinations of biomarkers used for the DARA response panel in the red square. (C) Multivariate ROC curve for the MM panel with the 95% CI shown in blue. (D) The average of predicted class probabilities of each sample across the 100 cross‐validations.

DARA has been shown to improve MM patient outcomes over the past decade; however, the majority of patients eventually relapse (Spencer et al. 2024), which is evident by only 10/57 patients in this study having a long‐term response to DARA. In order to evaluate the predictive efficacy of PB EV markers ROC curves were plotted, and AUC values for the PB EV markers ranged between 0.501 and 0.859 (Table 3), with the top five markers overlaid for comparison (Figure 6A).

**TABLE 3:** Univariate ROC curve statistics for long‐term DARA responders relative to short‐term responders and non‐responders.

Name	AUC	*t*‐tests	Log2 FC
**PDL1**	0.85882	**0.0001**	−1.7273
**LAT1**	0.85294	**0.0263**	−0.2011
**CD8**	0.60735	**0.0330**	−0.432
**CD44**	0.53824	0.1531	−0.2413
**CD61**	0.51176	0.1628	−0.5835
**CD38**	0.69118	0.2894	−0.3833
**CD63**	0.54412	0.4407	−0.182
**CD147**	0.64412	0.4580	0.06303
**CD31**	0.50147	0.5436	−0.0931
**CD29**	0.55147	0.5563	−0.1569
**CD36**	0.58235	0.5946	−0.2301
**CD321**	0.50588	0.7082	−0.0532
**CD9**	0.55882	0.7337	−0.1264
**CD59**	0.50588	0.7464	−0.0623
**CD81**	0.51471	0.8589	0.05257
**CD55**	0.56176	0.8655	0.04588

The strongest combination of PB EV markers (Response panel; CD55, LAT1 and PDL1) were selected based on the marker's frequency of being selected in the best classification model; however, the panel performed better with CD38 removed (Figure 6B). Multivariate ROC curves revealed that the response panel has a AUC of 0.733 [95% CI 0.123–0.969, *p* = 0.028; Figure 6C], which is slightly lower than the best performing individual marker PDL1 [AUC of 0.746, 95% CI 0.012–0.963, *p* = 0.031; Figure S5A]; however, it should be noted the confidence interval is very wide for both indicating the estimate is not very precise. This is most likely due to a combination of a low number of long‐term responders and a lower number of markers with a strong correlation with response. With this limitation affecting the measurement of the AUC value, we next examined how PDL1 and the response panel performed at classifying the patients correctly. The response panel correctly classified eight of 10 long‐term responders, and 31 of 34 short‐term responders and non‐responders into their respective groups (sensitivity 80% and specificity 91.2%), resulting in an overall accuracy of 88.6% (Figure 6D, Table 4), which is an improvement on PDL1 accuracy alone of 84.1% (Figure S5B, Table 4).

**TABLE 4:** DARA response panel versus PDL1 confusion matrix.

	DARA response panel		PDL1	
	Negative	Positive	Negative	Positive
**Long‐term responders**	2	8	2	8
**Short‐term responders and non‐responders**	31	3	29	5
**Accuracy**	88.6%		84.1%	
**Sensitivity**	80%		80%	
**Specificity**	91.2%		85.3%	
**Positive PV**	72.7%		61.5%	
**Negative PV**	93.9%		93.5%	

## 4 Discussion

EVs are important biomarker candidates with potential as a minimally invasive way to diagnose and monitor disease, and have been suggested to contribute to the generation of therapeutic resistance, suppression of the immune system and promotion of cancer progression (De Luca et al. 2019; Aung et al. 2011; Shah et al. 2018; Ciravolo et al. 2012). We have previously shown eight markers to be present on all MM patient PB EVs tested, with CD55, CD59 and CD147 being present at significantly higher levels relative to healthy controls (Brennan et al. 2022). In this study we expanded that list to 16 and identified five new proteins CD8, CD31, CD36, CD44 and LAT1, whose protein levels were significantly altered between MM patients and healthy control PB EVs. It is not known whether circulating PB EVs from MM patients can reflect the phenotype of myeloma cells residing in the BM that are usually characterized following a BM aspirate and/or biopsy; however, we have previously shown that CD38 and PDL1 levels were similar between PB and BM EVs. Furthermore, we had found CD59 and CD147 to be elevated in MM patient PB relative to healthy control PB and the levels of CD59 and CD147 were even higher in MM BM EVs (Brennan et al. 2022). In this report, we identified that CD44 follows a similar pattern of expression as CD59 and CD147 and was higher in MM BM EVs than PB EVs; however, CD8 showed the opposite correlation with MM PB EVs having less CD8 than healthy controls, while CD8 was significantly higher in the BM. We hypothesise that thePB EV populations are a mix of myeloma EVs, EVs from the immune system indicating the patient's response to the disease as well as EVs from other cells in the body. Since the myeloma cells are more abundant in the BM we hypothesise that there would be a higher percentage of tumour EVs located in BM than in the PB EV population. Therefore, if CD44, CD59 and CD147 positive EVs are derived from myeloma cells then it would explain the finding that these proteins are higher in the BM EV population than the PB EV population. On the other hand, CD8 is a T cell marker and these EVs may indicate that there are more T cells in the BM than in circulation in MM patients. A limitation to the study is that we do not have BM samples from healthy controls or DARA responders due to lack of ethical approval for sample collection, so it can't be determined whether CD8 is elevated in the BM EVs of MM patients relative to healthy controls. Interestingly LAT1 levels followed the same pattern as CD8, although it was not significantly elevated in the BM. It is not clear what cell type LAT1 originates from as LAT1 has been reported to play a role in T cell activation (Ogbechi et al. 2023), while other reports show LAT1 overexpression being significantly associated with high proliferation and poor prognosis in newly diagnosed MM (NDMM) patients (Isoda et al. 2014). CD44 was also significantly higher in DARA treated MM patients than NDMM patients, which was a similar pattern as observed for CD55 and CD59 (Brennan et al. 2022); however, this observation was from a group of NDMM containing only four patients and would require a larger study to validate this observation. CD44 has been previously linked with mediating resistance to lenalidomide in MM (Bjorklund et al. 2014) and Harshman et al. (2016) reported that newly diagnosed MM patients with >280 ng/mL serum CD44 had reduced overall survival, with serum EV depletion experiments showing that CD44 is primarily localized to the peripheral EVs in these patients (Harshman et al. 2016). CD31 and CD36 were significantly increased in MM PB EVs relative to healthy controls; however, there was no significant change between PB and BM. Our results highlight that only two of the eight proteins were significantly altered in the BM EVs of myeloma patients not responding to DARA relative to the PB EVs from the same patients. Overall, our data suggests that there is a high degree of similarity between PB and BM matched pairs and supports the use of circulating EV as a counterpart of the BM EV proteome as a liquid biopsy, and which is consistent with an observation made by Ferreira et al. (2022).

Previously only PDL1 was associated with initial DARA response (Brennan et al. 2022); however, there is now 2+ years additional follow‐up on the patient cohort, which allowed these patients to be subdivided into long‐term responders and short‐term responders. We found that three proteins (CD38, LAT1 and PDL1) were associated with a long‐term response to DARA. Hierarchical clustering of the protein markers revealed three main protein clusters, cluster 1 was higher in healthy and long‐term responders, while clusters 2 and 3 were lower in healthy controls. Furthermore, PCA revealed it was possible to distinguish between healthy PB EVs from MM PB EVs, and long‐term responders from other DARA treated patients. Using these groupings as a starting point we developed two signatures; a MM panel (CD8, CD44, CD147 and PDL1) that can distinguish between healthy PB EVs from MM PB EVs, and a DARA response panel (CD55, LAT1 and PDL1) that can identify long‐term responders from other DARA treated patients. The MM panel had a higher overall accuracy of 87.5% which is an improvement on CD147 accuracy alone of 71.4%. The response panel and the best performing individual marker PDL1, both had the same sensitivity for identifying long‐term responders (80%); however, the response panel was slightly more specific and correctly classified more short‐term responders and non‐responders, resulting in an overall accuracy of 88.6%, which is an improvement of PDL1 accuracy alone of 84.1%. The myeloma marker CD38 was independently associated long‐term response to DARA, but was not included in the response panel as combinations of PDL1, CD38; and CD38, PDL1, LAT1, performed worse than PDL1 alone (Figure S6); however, a larger number of long‐term responders are needed to validate which combination has the greatest accuracy.

Between this study and our previous study, we have identified for the first time that several proteins are present on PB and BM EVs from myeloma patients, with the levels of some proteins being significantly changed from healthy controls. We identified a MM panel of biomarkers which had a 16% better accuracy at identifying DARA‐treated MM patients compared to any of the biomarkers individually. We also observed several proteins changing in patients responding to a DARA‐containing regimen, with the response panel having a 4.5% better accuracy at identifying long‐term response to DARA compared to any of the biomarkers individually. Although the response panel provided a small improvement in this study, the analysis of a wider panel in a larger number of patients could potentially improve performance. This highlights the potential of EV based liquid biopsy; however, further studies will be needed to overcome the limits of this study in order to develop an assay that can be translated to the clinic. This study involved 57 myeloma patients undergoing a DARA‐containing regimen and 12 healthy controls and increasing the number of patients involved would improve the study's statistical power and improve the accuracy of the biomarker panel evaluation. In this study we relied on cross validation within the study population since we didn't have a separate validation cohort, therefore these findings need independent validation. Furthermore, prior to clinical translation the methodology may also need to be adapted from the EV focused ‘discovery’ methods (ultracentrifugation, density gradient ultracentrifugation, nanoflow cytometry EV counting and EV‐Bead conjugated flow cytometry) used in this study to methods such as EV‐Bead capture, ELISA or nano flow cytometry which would be more applicable to clinical use as they have a higher throughput and shorter turnaround time. The discovery methods we used offer improved separation of EVs from non‐EV components but have a lower EV yield than other methods and have a turnaround time of 3–4 days from blood sample to EV protein expression result, with ultracentrifuge rotors having a limit of six tubes at a time limiting sample throughput.

In conclusion, PB EVs are a source of non‐invasive biomarkers with potential for routine monitoring and to complement or replace invasive BM transplant. In this study, we expanded on the observations from our previous study to identify eight additional proteins expressed in all MM PB and BM EV samples tested. Five of these proteins (CD8, CD31, CD36, CD44 and LAT1) were significantly different from healthy controls and may have diagnostic value in the form of our proposed MM panel. Three proteins (CD38, LAT1 and PDL1) are associated with long‐term response and may have prognostic value in the form of our proposed DARA response panel. Further studies with longitudinal sampling are required to identify the most informative sampling time after initiation of therapy, to determine the maximum clinical benefit of the EV signatures identified through this study.

## Author Contributions

**Kieran Brennan**: conceptualization, investigation, methodology, writing—review and editing, writing – original draft, formal analysis, visualization, data curation. **Thomas Lund**: writing – review and editing. **Torben Plesner**: project administration, supervision, funding acquisition, writing – review and editing, conceptualization, resources. **Margaret M. Mc Gee**: supervision, funding acquisition, project administration, writing – review and editing, conceptualization, resources. **Katrine F. Iversen**: investigation, conceptualization, formal analysis, resources, writing – review and editing, funding acquisition. **Alfonso Blanco‐fernández**: formal analysis, methodology.

## Funding

This research was supported by the UCD Wellcome Institutional Strategic Support Fund, which was financed jointly by University College Dublin and the SFI‐HRB‐Wellcome Biomedical Research Partnership (ref. 204844/Z/16/Z); the TwinFlag consortium (HORIZON‐WIDERA‐2021‐ACCESS‐03‐01); 101079489, UCD Equip Funding Programme (ref 2021129) and Science Foundation Ireland Research Infrastructure Programme (ref 21/RI/9718). Furthermore, we received grants from Holms Mindelegat (20034), The Region of Southern Denmark (Region Syddanmarks Forksningspulje 2019 19/12124/A233), and the Dagmar Marshalls Foundation (500020), and a private donation from Lars‐Erik Houmann Christensen.

## Conflicts of Interest

The authors declare no conflict of interest. The funders had no role in the design of the study; in the collection, analyses, or interpretation of data; in the writing of the manuscript, or in the decision to publish the results.

## Supporting information



Supplementary Materials: jev270213‐0001‐SuppMat.docx

Supplementary Materials: jev270213‐0002‐SuppMat.pdf

Supplementary Materials: jev270213‐0003‐SuppMat.xlsx

## Data Availability

The data that support the findings of this study are available from the corresponding author upon reasonable request.
