# Supplementary material for: Identification of Extracellular Vesicle Signatures of Daratumumab Treated Multiple Myeloma
Source: J Extracell Vesicles. 2026 Feb 8;15(2):e70213. doi: 10.1002/jev2.70213 (PMC12883999; doi:10.1002/jev2.70213)
Supplement: Supplementary file 1 — Supplementary Materials: jev270213‐0001‐SuppMat.docx [file JEV2-15-e70213-s003.docx]

Supplemental data

**Supplemental Table 1. Patient characteristics.**

| **Patient Characteristics** | **Responders (n = 19)** | **Non-Responders (n = 38)** |
| --- | --- | --- |
| Age *; years; median (range) | 61 (49–83) | 66 (47–84) |
| Sex; n (%) |  |  |
| Female | 7 (37) | 20 (53) |
| Male | 12 (63) | 18 (47) |
| Immunoglobulin subtype *; n (%) |  |  |
| IgG | 12 (63) | 25 (66) |
| IgA | 1 (5) | 3 (8) |
| Light-chain only | 6 (32) | 9 (23.5) |
| Non secretory | 0 (0) | 1 (2.5) |
| ISS *; n(%) |  |  |
| I | 5 (26) | 13 (34) |
| II | 8 (42) | 8 (21) |
| III | 4 (21) | 5 (13) |
| Unknown | 2 (11) | 12 (32) |
| ECOG performance status *; n (%) |  |  |
| 0 | 9 (47) | 20 (53) |
| 1 | 7 (37) | 6 (16) |
| 2 | 0 (0) | 0 (0) |
| 3 | 0 (0) | 2 (5) |
| Unknown | 3 (16) | 10 (26) |
| Fluorescence in situ hybridization ^§^; n (%) |  |  |
| High-risk | 1 (5) | 9 (24) |
| Standard-risk | 15 (79) | 19 (50) |
| Unknown | 3 (16) | 10 (26) |
| Number of prior lines of therapy; median (range) | 2 (1–5) | 4 (0–17) |

* = at the time of diagnosis of multiple myeloma. § = If assessed more than once, the most recent result prior to initiation of DARA treatment is shown. High-risk aberrations were defined by the presence of either t(4;14), t(14;16) or del17p, each detected with a cut-off of 10% according to national standards for cytogenetic evaluation [45].

**Supplemental Table 2. Number of patient EV samples analysed by flow cytometry for each protein**

| **Protein**  **name** | **Total MM**  **Patients** | **Non-responder** | **Responder** | **Long-term responder** | **short-term responder** | **Healthy** | **NDMM** |
| --- | --- | --- | --- | --- | --- | --- | --- |
| **CD63** | **61** | **38** | **17** | **12** | **6** | **12** | **4** |
| **CD147** | **61** | **38** | **17** | **12** | **6** | **12** | **4** |
| **CD38** | **61** | **38** | **17** | **12** | **6** | **12** | **4** |
| **CD55** | **61** | **38** | **17** | **12** | **6** | **12** | **4** |
| **PDL1** | **61** | **38** | **17** | **12** | **6** | **12** | **4** |
| **CD59** | **60** | **37** | **17** | **12** | **6** | **12** | **4** |
| **CD9** | **58** | **35** | **17** | **12** | **6** | **12** | **4** |
| **CD81** | **57** | **35** | **17** | **11** | **6** | **12** | **4** |
| **CD44** | **54** | **33** | **17** | **11** | **6** | **12** | **4** |
| **LAT1** | **53** | **32** | **17** | **11** | **6** | **12** | **4** |
| **CD8** | **53** | **32** | **17** | **11** | **6** | **12** | **4** |
| **CD36** | **50** | **31** | **15** | **10** | **5** | **12** | **4** |
| **CD61** | **50** | **31** | **15** | **10** | **5** | **12** | **4** |
| **CD29** | **50** | **31** | **15** | **10** | **5** | **12** | **4** |
| **CD31** | **50** | **31** | **15** | **10** | **5** | **12** | **4** |
| **CD321** | **49** | **30** | **15** | **10** | **5** | **12** | **4** |

**Supplemental Table 3. Minimum Information about a Flow Cytometry Experiment (MIFlowCyt) checklist**

| **Requirement** | **Please Include Requested Information** |
| --- | --- |
| 1.1. Purpose | To compare the abundance of CD8, CD29, CD31, CD36, CD44, CD61, CD321 and LAT1 in plasma and bone marrow EV samples from healthy controls or multiple myeloma patients undergoing Daratumumab therapy. |
| 1.2. Keywords | EV; extracellular vesicles, Daratumumab, multiple myeloma , CD8, CD29, CD31, CD36, CD44, CD61, CD321 and LAT1 |
| 1.3. Experiment variables | Healthy controls or multiple myeloma patient plasma and bone marrow EVs were isolated by density gradient ultracentrifugation. Violet scatter-based triggering was used for the detection and counting of particles (VSSC gain=96; VSSC-H threshold=3000). Parameters detected: violet side-scatter (VSSC), red side-scatter (RSSC).  1.25 x 10^7^ EVs/test were bound to the surface of 4µm aldehyde/sulfate latex beads and stained with antibodies for the CD8, CD29, CD31, CD36, CD44, CD61, CD321 and LAT1for 30 min on ice. The samples were then resuspended in 200 µl PBS and flow cytometry analysis was performed on a Beckman Coulter CytoFLEX LX Flow Cytometer. Gating of EV-decorated 4 μm in diameter beads was performed based on FCS/SSC parameters, so that unbound EVs or possible antibody aggregates are excluded from the analysis. |
| 1.4. Organization name and address | Conway Institute, University College Dublin, Belfield, Dublin 4, Ireland |
| 1.5. Primary contact name and email address | Margaret Mc Gee, margaret.mcgee@ucd.ie  Kieran Brennan, k.brennan@ucd.ie |
| 1.6. Date or time period of experiment | May 2020 and June 2024. |
| 1.7. Conclusions | The level of CD31, CD36 and CD44 are elevated on MM PB EVs while CD8 and LAT1 levels are decreased compared with healthy controls and the level of CD38, LAT1 and PDL1 on MM PB EVs is higher in patients with a long-term response to Daratumumab, while CD44 is elevated in Daratumumab treated patients relative to newly diagnosed Multiple Myeloma patients. Multiple Myeloma bone marrow EVs have elevated CD8 and CD44 levels compared to peripheral blood EV. |
| 1.8. Quality control measures | Daily calibration of the flow cytometer with Beckman Coulter CytoFLEX Daily QC and CytoFLEX Daily IR QC Fluorospheres beads as per manufacturer specifications, followed by Apogee Mix 1527 Silica (Si) and polystyrene (PS) beads (Apogee Flow Systems Ltd. Hertfordshire, UK) were used in sizes of PS 80nm, PS 110nm, Si 180nm, Si 240nm, Si 300nm, PS 500nm, Si 590nm, Si 880nm and Si 1300nm. |
| 2.1.1.1. (2.1.2.1., 2.1.3.1.) Sample description | Healthy controls or multiple myeloma patient plasma and bone marrow EVs were isolated by density gradient ultracentrifugation |
| 2.1.1.2. Biological sample source description | Platelet free plasma (PFP) was obtained by centrifuging peripheral blood (PB) and bone marrow (BM) aspirate samples two times at 2,500×g at 4°C, for 15 min. Samples were stored at -80°C until EV isolation by density gradient ultracentrifugation – see Materials and Methods section for description. |
| 2.1.1.3. Biological sample source organism description | Fifty-seven patients diagnosed with MM according to the IMWG guidelines and treated with a DARA-containing regimen at the Departments of Haematology at either Vejle Hospital or Odense University Hospital, Denmark participated in the study (29). Additionally, four patients with untreated, newly diagnosed multiple myeloma (NDMM) and twelve healthy subjects were included as controls. The heathy donors were matched by age and sex with the MM population. Participation was voluntarily, and written informed consent was obtained from all subjects. Samples were obtained between December 2019 and May 2021 – see Materials and Methods section for description. |
| 2.1.2.2. Environmental sample location | NA |
| 2.3. Sample treatment description | Healthy controls or multiple myeloma patient plasma and bone marrow EVs in PBS were serially diluted from 1:2 to 1:500 to achieve an event count of 5000 events/s (30,000 events/µl) and gated using the gating strategy in Figure S2. Gating Strategy for extracellular (EVs) identification. The EV count was determined as the events/µl within the microparticle region multiplied by the dilution factor.  For EV-bead conjugated flow cytometry, 1.25 x10^7^ EVs/test was mixed with 0.2 µl/test aldehyde/sulfate latex beads (4 μm; Thermo Fisher Scientific, Waltham, USA) in 200 µl PBS rotating overnight at 4^o^C, with beads without EVs being used as a negative control. To block non-specific protein binding to beads 200 µl 2% BSA (2% BSA, 2 mM EDTA, 0.1% sodium azide in PBS) was then added to the samples to a final volume of 400 µl for 1h at RT, followed by 45 µl of 1M glycine for 30 mins at RT. The samples were then centrifuged at 5500 g for 5 min, the supernatant was removed and the beads were resuspended in 100 µl PBS and 2 µl FC block was added for 10 mins at RT. The samples were then centrifuged at 5500 g for 5 min and washed with 500 µl PBS three times. The beads were resuspended in 1% BSA 100 µl/test and aliquoted into fresh tubes. The beads were stained with antibodies for 30 min on ice and then centrifuged at 5500 g for 5 min and washed with 500 µl PBS three times. The samples were then resuspended in 200 µl PBS and flow cytometry analysis was performed on the Beckman Coulter CytoFLEX LX Flow Cytometer. Gating of EV-decorated 4 μm diameter beads was performed based on FCS/SSC parameters, so that unbound EVs or possible antibody aggregates are excluded from the analysis. |
| 2.4. Fluorescence reagent(s) description | Anti-CD8-PE-CY7 (1–25, clone RPA-T8, 25-0088-42 Thermo Fisher Scientific (Waltham, MA, USA)), anti-CD9 mouse-IgG1-PE (1-25, clone M-L13, 555372 BD Bioscience, Lot No.9337241), anti-CD29-PE (1–100, clone TS2/16, 568729, BD Bioscience), anti-CD31-PE (1–100, clone WM59, 555446 BD Bioscience), anti-CD36-PE (1–200, clone TR9, MA119772 Thermo Fisher Scientific (Waltham, MA, USA)), anti-CD38-FITC (1-200, clone CYT-38F2, 1911229 CYTOGNOS, Lot No. 2011658), anti-CD44-PE (1–200, clone 515, 550989 BD Bioscience), anti-CD55 mouse-IgG2-BV750 (1-200, clone A10, 750101 BD Bioscience, Lot No. 9267679), anti-CD59 mouse-IgG1-APC (1-200, clone OV9A2, 17-0596-42 Thermo Fisher Scientific, Lot No. 2213390), anti-CD61-PE (1–200, clone VI-PL2, 555754 BD Bioscience), anti-CD63 mouse-IgG1-PE (1-100, clone H5C6, 12-0639-42 Thermo Fisher Scientific, Lot No. 2124708), anti-CD81 mouse-IgG1-APC (1-25, clone JS-81, 551112 BD Bioscience, Lot No.9311678), anti-CD147 mouse-IgG1-APC (1-400, clone MEM-M6/1, A15706 Thermo Fisher Scientific, Lot No. 76056999), anti-CD321-PE (1–200, clone M.Ab.F11, 552556 BD Bioscience), anti-LAT1-PE (1–100, clone BU53, NBP2-50465PE, Novus Biologicals), anti-PD-L1 mouse-IgG1-PE-CY7 (1-100, clone MIH1, 558017 BD Bioscience, Lot No. 8312928), IgG1 isotype control-FITC (1-125, clone MOPC-21, 554679 BD Bioscience, Lot No. 9199419), IgG1 isotype control-PE (1–125, clone MOPC-21, 559320 BD Bioscience), and IgG2 -BV750 (1-200, clone G155-178, 553456 BD Bioscience, Lot No. 0239705) were used to label healthy controls or multiple myeloma patient plasma and bone marrow EVs. |
| 3.1. Instrument manufacturer | Beckman Coulter |
| 3.2. Instrument model | Beckman Coulter CytoFLEX LX Flow Cytometer |
| 3.3. Instrument configuration and settings | CytoFLEX LX (100mW 405nm, 50mW 488nm, 50mW 561nm, 50mW 638nm)  405 – 405/10 (V-SSC), 488 – 488SSC (B-SSC), 488 – 530/30 (Apogee beads FITC), 638 – 638/6 (R-SSC), 638 – 660/10 (APC) |
| 4.1. List-mode data files | FCS files and the analysis workspace have been uploaded data repository and can also be obtained by contacting the corresponding author. |
| 4.2. Compensation description | No compensation was needed |
| 4.3. Data transformation details | No transformation was needed |
| 4.4.1. Gate description | See Methods section and Figure S1 of manuscript |
| 4.4.2. Gate statistics | See Methods section, Figure S1 and Table S5 of manuscript |
| 4.4.3. Gate boundaries | See Methods section and Figures S1 of manuscript |

**Supplemental Table 4. Minimum Information about a Flow Cytometry Experiment (MIFlowCyt) EV Reporting Framework**

| **Framework Criteria** | **Please complete each criterion** |
| --- | --- |
| 1.1 Preanalytical variables conforming to MISEV guidelines. | Fifty-seven patients diagnosed with MM according to the IMWG guidelines and treated with a DARA-containing regimen at the Departments of Haematology at either Vejle Hospital or Odense University Hospital, Denmark participated in the study (29). Additionally, four patients with untreated, newly diagnosed multiple myeloma (NDMM) and twelve healthy subjects were included as controls. The healthy donors were matched by age and sex with the MM population. Participation was voluntary, and written informed consent was obtained from all subjects. Samples were obtained between December 2019 and May 2021 – see Materials and Methods section for description.  Platelet free plasma (PFP) preparation  Platelet free plasma (PFP) was obtained by centrifuging peripheral blood (PB) and bone marrow (BM) aspirate samples two times at 2,500×g at 4°C, for 15 min. Samples were stored at -80°C until EV isolation.  Ultracentrifugation (UC) of PB plasma  PB PFP samples were defrosted and diluted with particle-free PBS before centrifugation at 2500 g for 15 min in an SX4250 rotor to pellet cell fragments and other debris. The supernatant was transferred to a 38 ml ultracentrifuge tube (Prod. No. 344058). The tubes were centrifuged at 120,000 g (RCF AVG, 31300 rpm) for 2 hours 40 min at 20 °C, using a SW32ti rotor. The supernatant was removed and the EV pellet was resuspended in 1 ml residual PBS and transferred to a 1 ml ultracentrifuge tube (Prod. No. 343778). The tubes were centrifuged at 120,000 g (RCF AVG, 51000 rpm) for 50 min at 20 °C, using a MLA130 rotor. The supernatant was removed and the EV pellet was resuspended in 200 µl residual PBS.  Ultracentrifugation (UC) of BM plasma  BM PFP samples were defrosted and diluted with particle-free PBS before being centrifuged at 2500 g for 15 min in an SX4250 centrifuge to pellet cell fragments and other debris. The supernatant was transferred to a 13 ml ultracentrifuge tube (Prod. No. 344059). The tubes were centrifuged at 120,000 g (RCF AVG, 31300 rpm) for 2 hours 45 min at 20 °C, using a SW41ti rotor. The supernatant was removed and the EV pellet was resuspended in 200 µl residual PBS.  Floatation density gradient (DG-UC)  Density gradient centrifugation was performed using a modified protocol from Brennan et al. A 54% iodixanol-PBS working solution (estimated density ~1.295 g/ml) was prepared by diluting a stock solution of OptiPrep™ (60% (w/v) aqueous iodixanol from Axis-Shield PoC, Norway) with 10x particle-free PBS (Gibco, Waltham, MA, USA). Iodixanol solutions (1.2 g/ml and 1.08 g/ml) were prepared by diluting the 54% iodixanol-PBS working solution in 1x particle-free PBS (Gibco, Waltham, MA, USA). To form the gradient, firstly a homogenous base layer of the gradient (estimated density ~1.224 g/ml) was produced by adding 672 µl of the 54% iodixanol-PBS working solution to a 13 ml ultracentrifuge tube (Prod. No. 344059), together with 200 µl either BM or PB PFP EVs isolated by ultracentrifugation. Next, 2 ml 1.2 g/ml iodixanol and 3 ml 1.08 g/ml iodixanol were layered successively on top of the vesicle suspension with the remainder of the tube filled with PBS. Centrifugation was performed at 197,120 g (RCF AVG) for 15 h at 4 °C in a SW41ti rotor (40,000 rpm). Fractions (~200 µl) were collected from the top of the tube. 50 µl of each fraction was pipetted into a 96 well plate and absorbance was measured at 340 nm against an iodixanol standard curve to determine the fraction density. The fractions with densities between 1.08–1.19 g/ml were combined and diluted to a density <1.03 g/ml with particle-free PBS and the diluted fractions were centrifuged at 120000 g (RCF AVG, 31300 rpm using a SW32ti rotor) for 3 hours 15 min at 20 °C. The supernatant was removed and the EV pellets were resuspended in 200 µl residual PBS and stored at -80°C prior to analysis. |
| 1.2 Experimental design according to MIFlowCyt guidelines. | 1.1 Purpose: To compare the abundance of CD8, CD29, CD31, CD36, CD44, CD61, CD321 and LAT1 in plasma and bone marrow EV samples from healthy controls or multiple myeloma patients undergoing Daratumumab therapy.  1.2 Keywords: EV; extracellular vesicles, Daratumumab, multiple myeloma , CD8, CD29, CD31, CD36, CD44, CD61, CD321 and LAT1  1.3 Experimental variables: Healthy controls or multiple myeloma patient plasma and bone marrow EVs were isolated by density gradient ultracentrifugation. Violet scatter-based triggering was used for the detection and counting of particles (VSSC gain=96; VSSC-H threshold=3000). Parameters detected: violet side-scatter (VSSC), red side-scatter (RSSC). |
| 2.1 Sample staining details | 1.25 x10^7^ EVs/test was mixed with 0.2 µl/test aldehyde/sulfate latex beads (4 μm; Thermo Fisher Scientific, Waltham, USA) in 200 µl PBS rotating overnight at 4°C, with beads without EVs being used as a negative control. To block non-specific protein binding to beads 200 µl 2% BSA (2% BSA, 2 mM EDTA, 0.1% sodium azide in PBS) was then added to the samples to a final volume of 400 µl for 1h at RT, followed by 45 µl of 1M glycine for 30 mins at RT. The samples were then centrifuged at 5500 g for 5 min, the supernatant was removed and the beads were resuspended in 100 µl PBS and 2 µl FC block was added for 10 mins at RT. The samples were then centrifuged at 5500 g for 5 min and washed with 500 µl PBS three times. The beads were resuspended in 1% BSA 100 µl/test and aliquoted into fresh tubes. The beads were stained with antibodies for 30 min on ice and in the dark and then centrifuged at 5500 g for 5 min and washed with 500 µl PBS three times. The samples were then resuspended in 200 µl PBS and flow cytometry analysis was performed on the Beckman Coulter CytoFLEX LX Flow Cytometer. Gating of EV-decorated 4 μm diameter beads was performed based on FCS/SSC parameters, so that unbound EVs or possible antibody aggregates are excluded from the analysis. |
| 2.2 Sample washing details | Washing steps were performed by centrifugation at 5500 g for 5 min and washed with 500 µl PBS three times. |
| 2.3 Sample dilution details | Samples were serially diluted from 1:2 to 1:500 to achieve an event count of 5000 events/s (30,000 events/µl) for EV counting. Samples were not diluted for EV-bead conjugated flow cytometry, instead the samples were resuspended in 200 µl PBS. |
| 3.1 Buffer alone controls. | A buffer-only control of 0.22 μm-filtered DPBS (Gibco 14190144) was recorded at the same flow cytometer acquisition settings as all other samples, including triggering threshold, and flow rate. The buffer-only control had a count of ~100 events/s.  For EV-bead conjugated flow cytometry, a buffer-only control of 0.22 μm-filtered DPBS (Gibco 14190144) with beads was used. |
| 3.2 Buffer with reagent controls. | Beads with buffer and reagent control (PBS and either anti-CD8-PE-CY7 (1–25, clone RPA-T8, 25-0088-42 Thermo Fisher Scientific (Waltham, MA, USA)), anti-CD9 mouse-IgG1-PE (1-25, clone M-L13, 555372 BD Bioscience, Lot No.9337241), anti-CD29-PE (1–100, clone TS2/16, 568729, BD Bioscience), anti-CD31-PE (1–100, clone WM59, 555446 BD Bioscience), anti-CD36-PE (1–200, clone TR9, MA119772 Thermo Fisher Scientific (Waltham, MA, USA)), anti-CD38-FITC (1-200, clone CYT-38F2, 1911229 CYTOGNOS, Lot No. 2011658), anti-CD44-PE (1–200, clone 515, 550989 BD Bioscience), anti-CD55 mouse-IgG2-BV750 (1-200, clone A10, 750101 BD Bioscience, Lot No. 9267679), anti-CD59 mouse-IgG1-APC (1-200, clone OV9A2, 17-0596-42 Thermo Fisher Scientific, Lot No. 2213390), anti-CD61-PE (1–200, clone VI-PL2, 555754 BD Bioscience), anti-CD63 mouse-IgG1-PE (1-100, clone H5C6, 12-0639-42 Thermo Fisher Scientific, Lot No. 2124708), anti-CD81 mouse-IgG1-APC (1-25, clone JS-81, 551112 BD Bioscience, Lot No.9311678), anti-CD147 mouse-IgG1-APC (1-400, clone MEM-M6/1, A15706 Thermo Fisher Scientific, Lot No. 76056999), anti-CD321-PE (1–200, clone M.Ab.F11, 552556 BD Bioscience), anti-LAT1-PE (1–100, clone BU53, NBP2-50465PE, Novus Biologicals), anti-PD-L1 mouse-IgG1-PE-CY7 (1-100, clone MIH1, 558017 BD Bioscience, Lot No. 8312928), IgG1 isotype control-FITC (1-125, clone MOPC-21, 554679 BD Bioscience, Lot No. 9199419), IgG1 isotype control-PE (1–125, clone MOPC-21, 559320 BD Bioscience), and IgG2 -BV750 (1-200, clone G155-178, 553456 BD Bioscience, Lot No. 0239705) were recorded at the same flow cytometer acquisition settings as all other samples, including triggering threshold, and flow rate. |
| 3.3 Unstained controls. | Unstained controls were measured at the same dilution as matched stained controls. Flow cytometer acquisition settings were maintained for all samples, including triggering threshold, and flow rate. |
| 3.4 Isotype controls. | IgG1 isotype control-FITC (1-125, clone MOPC-21, 554679 BD Bioscience, IgG1 isotype control-PE (1–125, clone MOPC-21, 559320 BD Bioscience), Lot No. 9199419), IgG2 -BV750 (1-200, clone G155-178, 553456 BD Bioscience, Lot No. 0239705) were used together with unstained controls were used as negative staining controls. |
| 3.5 Single-stained controls. | Not applicable, samples were either unstained or single stained. |
| 3.6 Procedural controls. | Controls and samples were acquired with the same gain settings, no compensation applied, low flow rate, stopping after recording the samples for 2 minutes. |
| 3.7 Serial dilutions. | Samples were serially diluted from 1:2 to 1:500 to achieve an event count of 5000 events/s (30,000 events/µl) and that dilution was used for antibody labelling. |
| 3.8. Detergent treated EV-samples | Not applicable |
| 4.1 Trigger Channel(s) and Threshold(s). | Based on the buffer alone control and Apogee Mix 1527 beads detection was triggered on the 405 nm laser (VSSC gain=96; VSSC-H threshold=3000). |
| 4.2 Flow Rate / Volumetric quantification. | The flow rate on the CytoFlex LX was calibrated with samples measured at 10 µl/min. |
| 4.3 Fluorescence Calibration. | Daily calibration of the flow cytometer CytoFLEX Daily QC and CytoFLEX Daily IR QC Fluorospheres beads (Beckman Coulter B53230 and C06147, respectively) as per manufacturer specifications. |
| 4.4 Light Scatter Calibration. | The gain of the three scatters was established with the help of the Apogee Mix beads (VSSC gain=96; VSSC-H threshold=3000), to minimize the noise and maximize the signal and trying to place the three scatters with very similar values. |
| 5.1 EV diameter/surface area/volume approximation. | Not preformed. |
| 5.2 EV refractive index approximation. | Not preformed. |
| 5.3 EV epitope number approximation. | Not preformed. |
| 6.1 Completion of MIFlowCyt checklist. | See attached MIFlowCyt Checklist. |
| 6.2 Calibrated channel detection range | Not applicable. |
| 6.3 EV number/concentration. | Healthy controls or multiple myeloma patient plasma and bone marrow EVs in PBS were serially diluted from 1:2 to 1:500 to achieve an event count of 5000 events/s (30,000 events/µl) and gated using the gating strategy in Figure S2. Gating Strategy for extracellular (EVs) identification. The EV count was determined as the events/µl within the microparticle region multiplied by the dilution factor. |
| 6.4 EV brightness. | Not applicable |
| 7.1. Sharing of data to a public repository. | FCS files and the analysis workspace have been uploaded data repository and can also be obtained by contacting the corresponding author. |

**Supplemental Table 5 Shortest of membrane proteins increased in MM PB EVs relative to healthy PB EVs**

| **Accession**  **No** | **Protein**  **ID** | **p-Value**  **Non-Responders vs Responders** | **p-Value**  **MM vs Healthy** | **Fold Change Non-Responders/Responders** | **Fold Change**  **MM vs Healthy** | **Increased in Cancer** |
| --- | --- | --- | --- | --- | --- | --- |
| P05556-2 | **CD29** | 0.008 | 0.017 | 8,106.83 | 4053.9 | + |
| P16284-5 | **CD31** | 0.011 | 0.044 | 6,414.80 | 8.9 | + |
| P01889 | HLA-B | 0.144 | 0.034 | 4.27 | 14.7 | - |
| Q01650 | **LAT1** | 0.194 | 0.039 | 4.24 | 1778.0 | + |
| P05106-2 | **CD61** | 0.047 | 0.074 | 2.65 | 2.1 | + |
| P35613-3 | **CD147** | 0.164 | 0.001 | 2.22 | 7782.7 | + |
| P08514-3 | CD41 | 0.249 | 0.005 | 2.02 | 10.2 | - |
| P16671-4 | **CD36** | 0.183 | 0.000 | 1.88 | 17.1 | + |
|  | | | | | | |
| P60033 | **CD81** | 0.509 | 0.033 | 0.59 | 7.0 | + |
| P13987 | **CD59** | 0.077 | 0.001 | 0.57 | 2.8 | + |
| P01732-2 | **CD8a** | 0.037 | 0.001 | 0.49 | 3.2 | - |
| P21730 | CD88 | 0.153 | 0.005 | 0.35 | 5226.1 | + |

Raw dataset is publicly available in the supplementary data of Brennan, et al. 2022 [11].

**
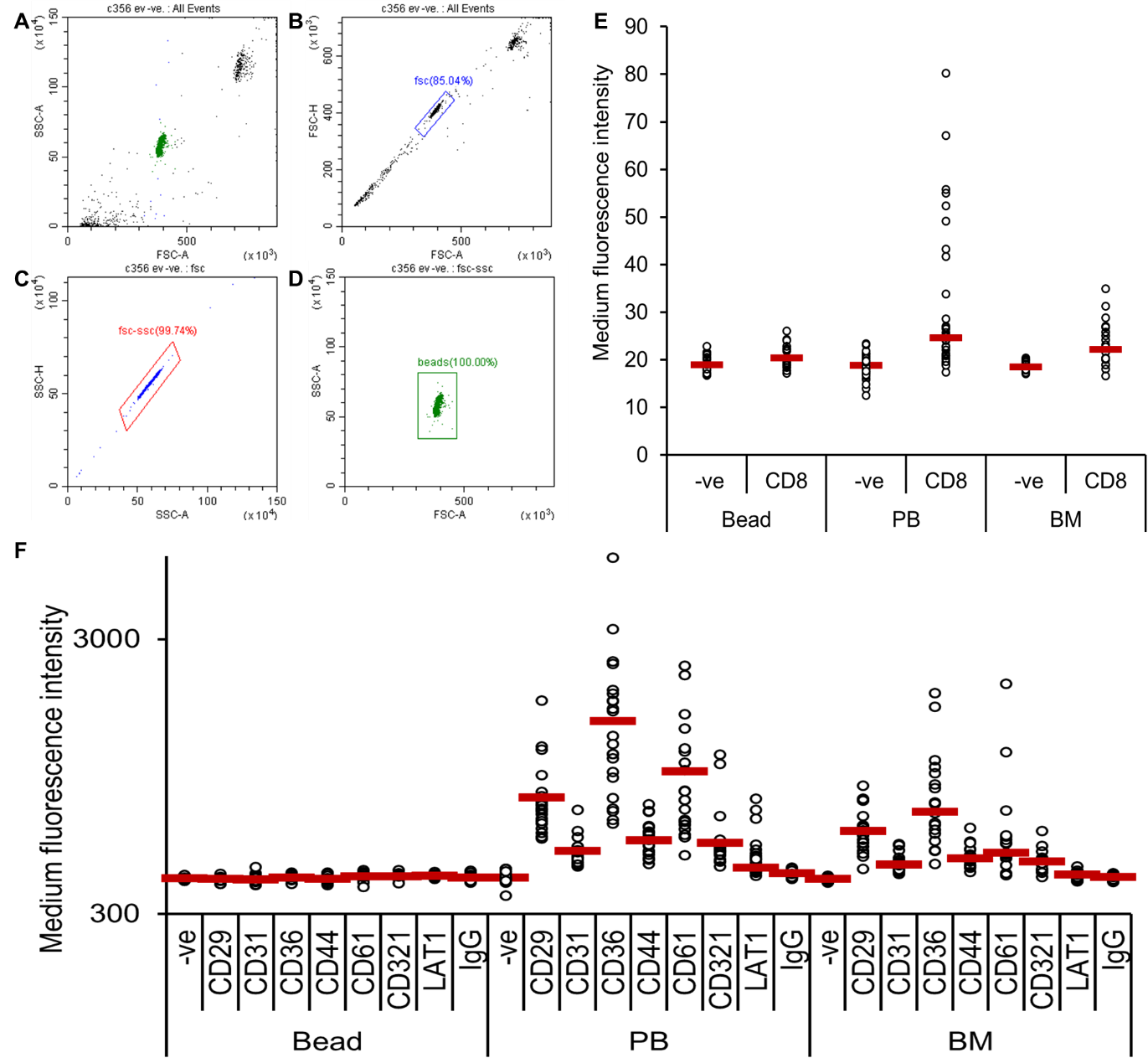
**

**Supplemental Figure 1. EV-bead gating and staining controls**

Aldehyde latex beads uncoated or coated with MM PB or MM BM EV samples and stained with antibodies, IgG1-PE isotype control, or unstained control (-ve) and recorded on a Cytoflex flow cytometer. **A**) The aldehyde latex beads region was defined on a SSC-A/ FSC-A dot-plot and to remove aggregates and debris the beads were identified within the FSC population on **(B**) a FSC-H/ FSC-A dot-plot. (**C**) The FSC events were then plotted on a SSC-H/ SSC-A dot-plot and events within the FSC-SSC region, which were then plotted on a SSC-A/ FSC-A dot-plot and **(D**) events within the bead region were used for analysis. Dot plots showed no non-specific binding to uncoated beads for **(E)** CD8-PE-CY7 and **(F)** CD29-PE, CD31-PE, CD36-PE, CD44-PE, CD61-PE, CD321-PE, LAT1-PE, IgG1-PE isotype control.

**
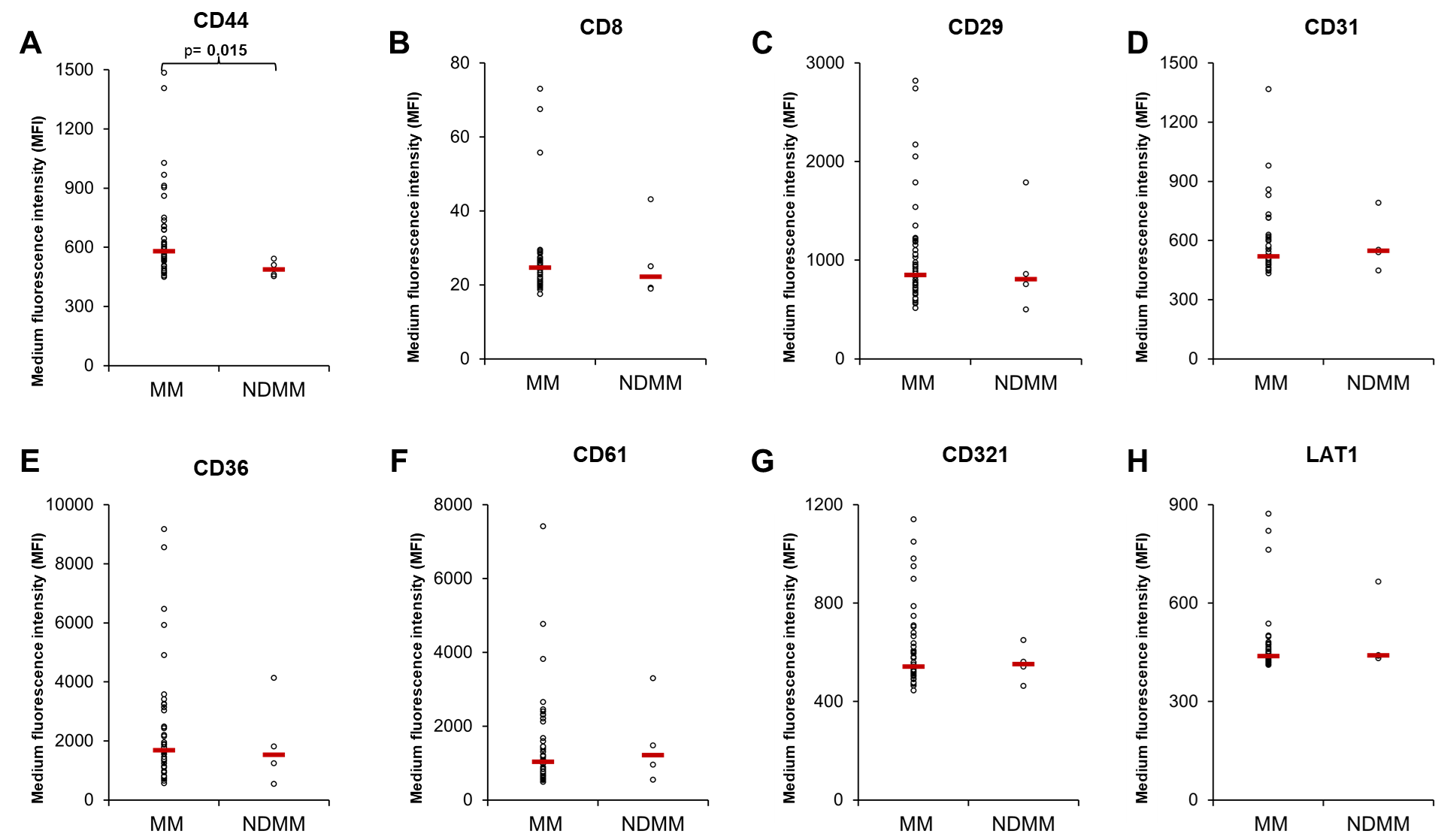
**

**Supplemental Figure 2 CD44 are higher on Multiple Myeloma (MM) peripheral blood EVs relative to newly diagnosed Multiple Myeloma (NDMM) PB EVs**

Flow cytometry analysis of 1.25 × 10^7^ EVs/test from 4 NDMM and 45-50 MM patients (the number of patients tested for each protein is listed in Table S2) were bound to the surface of 4 µm aldehyde/sulfate latex beads and stained with antibodies; CD8-PE-CY7, CD29-PE, CD31-PE, CD36-PE, CD44-PE, CD61-PE, CD321-PE and LAT1-PE for 30 min on ice.

**
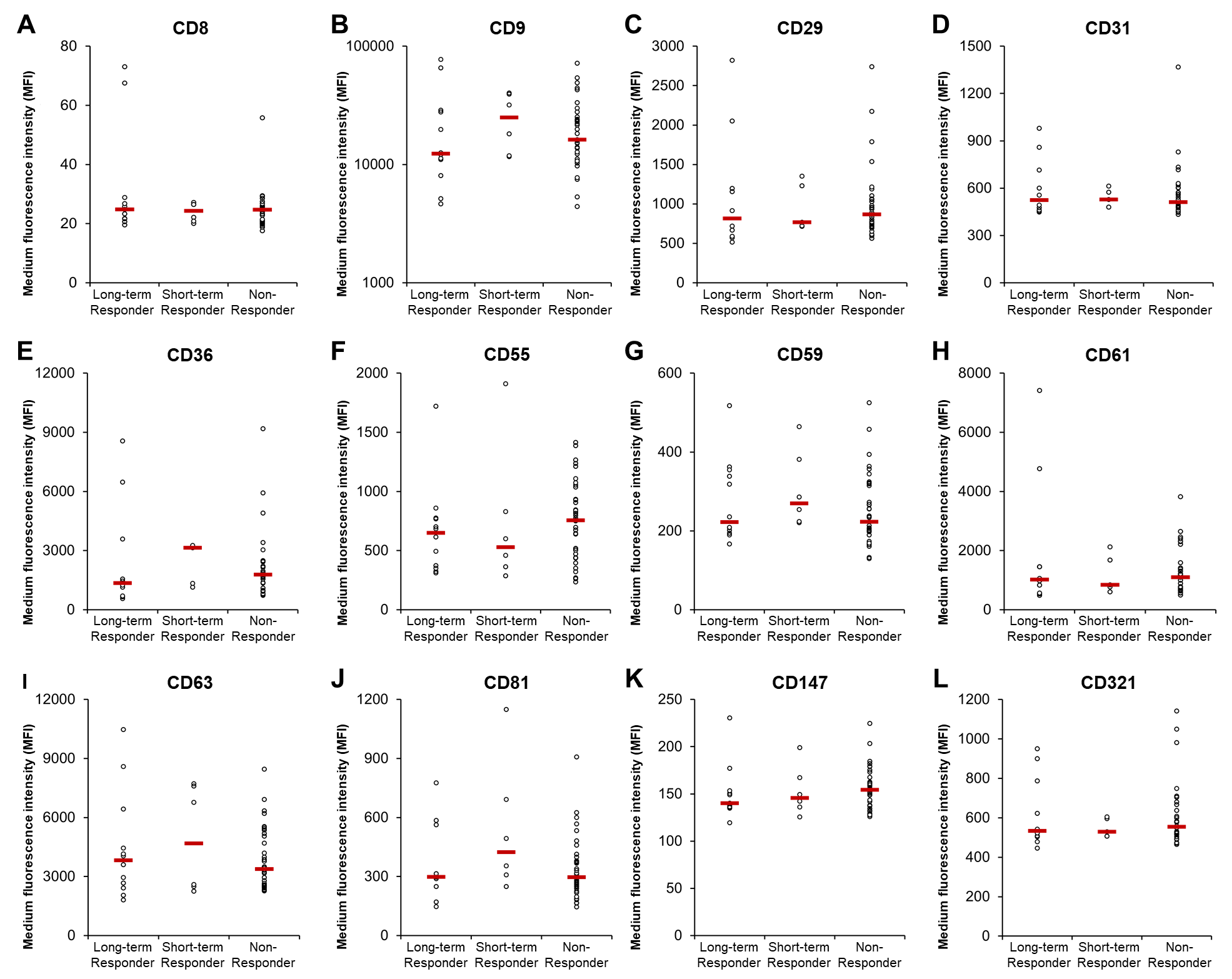
**

**Supplemental Figure 3. Protein expression relative to MM patient response to DARA**

Flow cytometry analysis of 1.25 × 10^7^ EVs/test from 10-12 long-term responders and 30-38 non-responders and 5-6 Short-term responders (the number of patients tested for each protein is listed in Table S2), were bound to the surface of 4 µm aldehyde/sulfate latex beads and stained with antibodies; CD8-PE-CY7, CD9-PE, CD29-PE, CD31-PE, CD36-PE, CD55-BV750, CD59-APC, CD61-PE, CD63-PE, CD81-APC, CD147-APC, and CD321-PE for 30 min on ice.

**
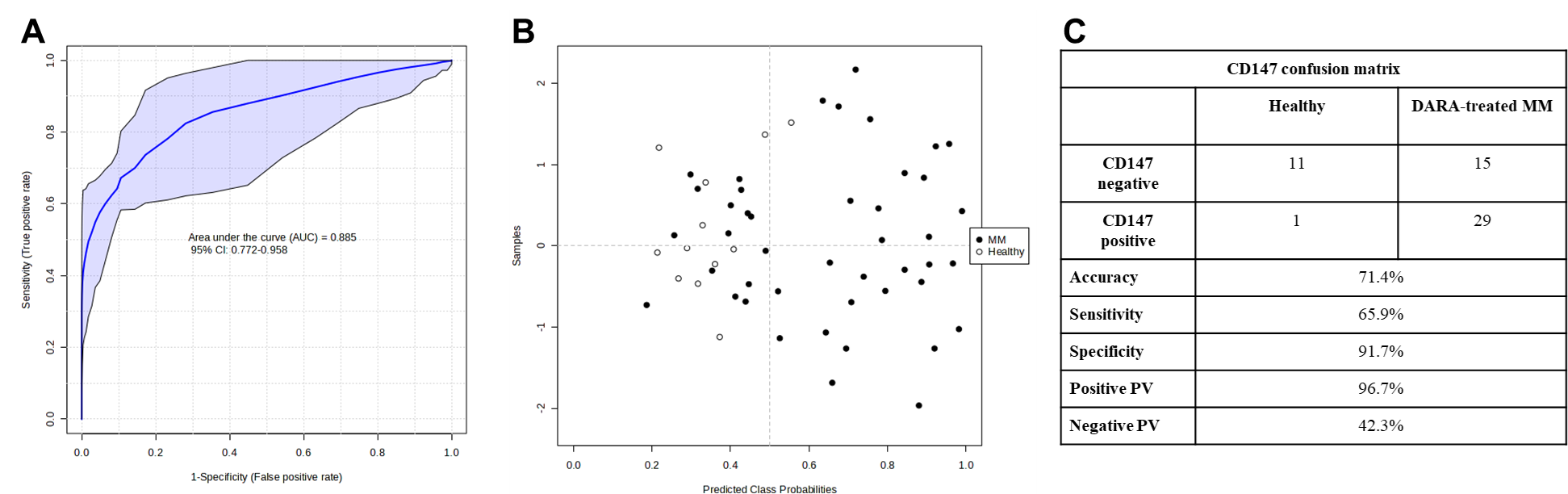
**

**Supplemental Figure 4. Evaluation of the diagnostic efficacy of CD147 to distinguish MM patients from healthy controls**

(A) Multivariate ROC curve for the CD147 with the 95% CI shown in blue. (B) The average of predicted class probabilities of each sample across the 100 cross-validations.

**
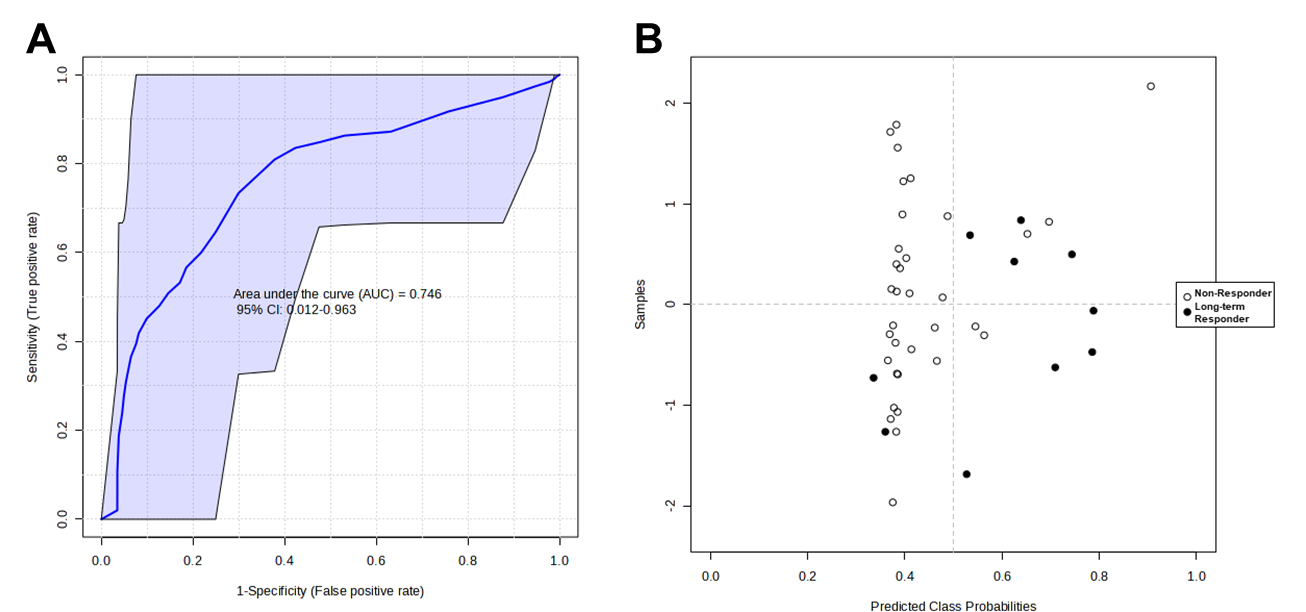
**

**Supplemental Figure 5. Evaluation of the predictive efficacy of the PDL1 to distinguish DARA Long-term Responders from Short-term Responders and Non-Responders**

(A) Multivariate ROC curve for the PDL1 with the 95% CI shown in blue. (B) The average of predicted class probabilities of each sample across the 100 cross-validations.

|  | **PDL1, CD38 p = 0.046** | |
| --- | --- | --- |
|  | negative | positive |
| Long-term Responders | 3 | 7 |
| Short-term Responders and Non-Responders | 28 | 6 |


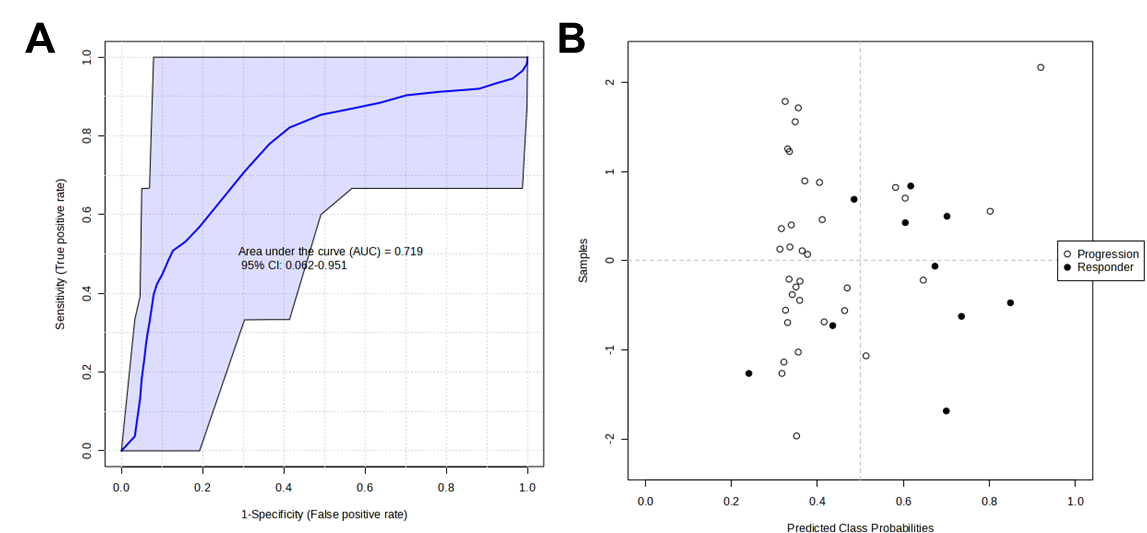


|  | **CD38, PDL1, LAT1 P=0.036** | |
| --- | --- | --- |
|  | negative | positive |
| Long-term Responders | 2 | 8 |
| Short-term Responders and Non-Responders | 28 | 6 |


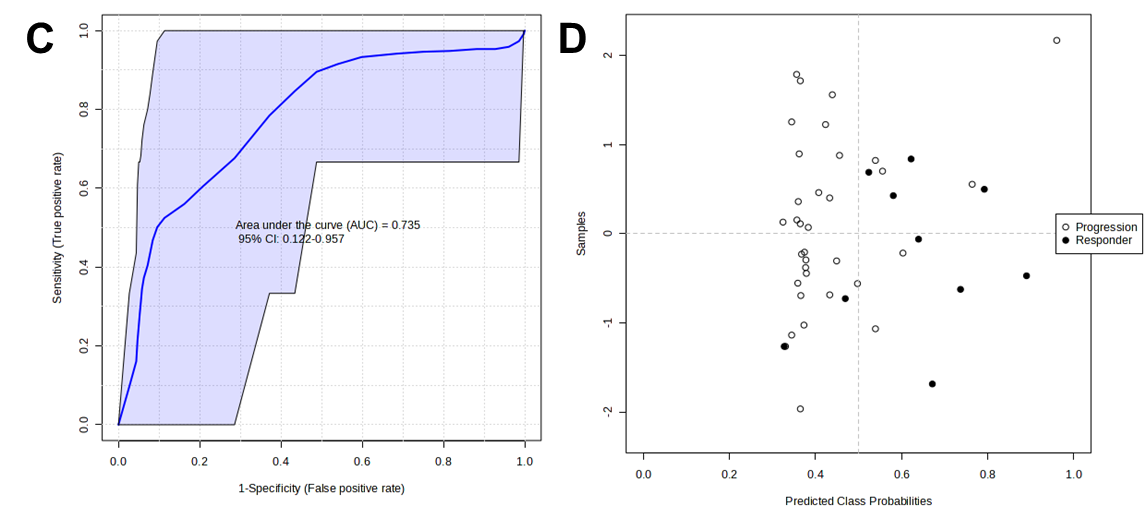


**Supplemental Figure 6. Evaluation of the predictive efficacy of a CD38 based response panel signatures to distinguish DARA Long-term Responders from Short-term Responders and Non-Responders**

The confusion matrix is shown for PDL1, CD38 with (A) showing the multivariate ROC curve for the PDL1, CD38 with the 95% CI shown in blue. (B) The average of predicted class probabilities of each sample across the 100 cross-validations. The confusion matrix is shown for CD38, PDL1, LAT1 with (C) showing the multivariate ROC curve for the CD38, PDL1, LAT1 with the 95% CI shown in blue. (D The average of predicted class probabilities of each sample across the 100 cross-validations.
